# Altered gut microbiota composition in adults with chronic subdural hematoma: a case-control study

**DOI:** 10.3389/fcimb.2026.1962090

**Published:** 2026-10-23

**Authors:** Yunfei Li, Xiaofan Yu, Chaoqun Lin, Guoyi Gao, Weiming Liu, Liang Wu

**Affiliations:** 1 Department of Neurosurgery, Beijing Tiantan Hospital, Capital Medical University, Beijing, China; 2 China National Clinical Research Center for Neurological Diseases, Beijing, China; 3 Department of Neurosurgery, Beijing Chaoyang Hospital, Capital Medical University, Beijing, China; 4 Department of Neurosurgery, Beijing Jishuitan Hospital, Capital Medical University, Beijing, China

**Keywords:** 16S rRNA sequencing, chronic subdural hematoma, gut microbiota, microbial diversity, random forest

## Abstract

**Background and purpose:**

Chronic subdural hematoma (CSDH) is increasingly recognized as a dynamic inflammatory and angiogenic disorder, but whether it is associated with alterations in the gut microbiota remains unclear. We therefore characterized the gut microbial profiles of adults with CSDH and healthy controls (HCs).

**Methods:**

We conducted an observational case-control study of 40 adults with CSDH and 96 HCs. Fecal bacterial communities were profiled by 16S rRNA gene sequencing. We assessed alpha and beta diversity, identified differentially abundant genera using covariate-adjusted centered log-ratio models with false-discovery-rate correction, evaluated genus-level discrimination using a random forest classifier with repeated nested cross-validation, and examined associations with clinical characteristics using partial Spearman correlations.

**Results:**

There was no clear evidence of an alpha-diversity difference after covariate adjustment. Community structure differed across Bray-Curtis, weighted UniFrac, and unweighted UniFrac distances (all P = 0.001), with the largest explained variance for weighted UniFrac (R² = 0.106). Thirteen genera remained significant after adjustment and multiple-testing correction, including sensitivity analyses that additionally accounted for diabetes and recorded medication exposure. *Collinsella* and *Dorea* were more abundant in CSDH, whereas *Bacteroides* was more abundant in HCs. Across 10 repeated outer cross-validation partitions, the mean out-of-fold area under the receiver-operating-characteristic curve was 0.938 ± 0.010; averaging participant-level out-of-fold probabilities yielded an area under the curve of 0.951. *Dorea* was positively associated with diabetes, and *Bacteroides* was inversely associated with cerebral infarction after false-discovery-rate correction.

**Conclusions:**

Adults with CSDH showed differences in gut microbial community structure and genus-level composition, without clear evidence of an alpha-diversity difference. These are exploratory associations in a selected case-control sample. The cross-sectional, single-center design precludes causal inference, and the classifier has not established calibration, external validity or clinical utility.

## Introduction

Chronic subdural hematoma (CSDH) is a common neurosurgical disorder that mainly affects older adults. Among people aged 70 years or older, incidence estimates reach 58 cases per 100,000 person-years (Kudo et al., 1992; Santarius and Hutchinson, 2004). As populations age, the clinical and healthcare burden of CSDH is expected to increase (Balser et al., 2015; Kolias et al., 2014). Population-level data have also linked rising subdural hematoma incidence with increasing antithrombotic drug use (Gaist et al., 2017).

CSDH was once regarded as a static blood collection but is now understood as a dynamic inflammatory and angiogenic process (Kolias et al., 2014). Local inflammation, fragile neovascularization, hyperfibrinolysis, and repeated microhemorrhage may contribute to hematoma enlargement and recurrence (Edlmann et al., 2017). The systemic factors associated with this inflammatory process remain poorly understood.

The gut microbiota contributes to immune homeostasis and metabolism (Belkaid and Hand, 2014; Lynch and Pedersen, 2016) and communicates bidirectionally with the nervous system through immune, neural, endocrine, and metabolic pathways (Cryan et al., 2019; Fung et al., 2017; Morais et al., 2021). Experimental studies further indicate that intestinal microbial perturbation can modify immune responses and outcome after cerebral ischemia (Benakis et al., 2016; Winek et al., 2016). Altered microbial profiles have also been reported in several human neurological conditions. Case-control studies have reported gut microbiota changes in moyamoya disease (Yu et al., 2023), mild cognitive impairment (Fan et al., 2023), and migraine with irritable bowel syndrome (Liu et al., 2022). However, findings vary across populations and analytical pipelines. The gut microbiota in adults with CSDH has not been systematically characterized.

We conducted a case-control study using 16S rRNA sequencing to characterize the gut microbiota in adults with CSDH. We compared microbial diversity and taxonomic composition between the CSDH and healthy control (HC) groups and identified covariate-adjusted differential genera. We also evaluated the internal discrimination of genus-level profiles and examined their associations with clinical characteristics. This cross-sectional study was designed to identify microbial signatures associated with CSDH, not to establish causality.

## Materials and methods

### Study population

This observational case-control study enrolled 40 adults with CSDH at the Department of Neurosurgery, Beijing Tiantan Hospital, Capital Medical University. Recruitment occurred between June 2023 and December 2024. The treating neurosurgeon diagnosed CSDH based on compatible clinical findings and a hypodense or isodense subdural collection on head computed tomography or brain magnetic resonance imaging. We also recruited ninety-six healthy controls (HCs) undergoing routine health examinations. None had evidence of CSDH on cranial computed tomography.

Eligible participants were older than 18 years, could provide a fecal sample, and had no autoimmune or gastrointestinal disorders. They had not used systemic hormonal medication, Chinese herbal medicine, antibiotics, probiotics, or prebiotics during the preceding 3 months. Participants had also not used nonsteroidal anti-inflammatory drugs (NSAIDs) during the 3 months before fecal sampling. We excluded pregnant or lactating participants. The Ethics Committee of Beijing Tiantan Hospital, Capital Medical University, approved the study (KY2023-265-02). All participants provided written informed consent before enrollment. We conducted the study in accordance with the Declaration of Helsinki and reported it according to the Strengthening the Reporting of Observational Studies in Epidemiology (STROBE) statement (von Elm et al., 2007).

CSDH-specific clinical descriptors comprised hematoma volume, midline shift and internal hematoma architecture. Postoperative recurrence within 180 days was recorded separately as a follow-up outcome. Hematoma volume was measured by slice-by-slice segmentation in 3D Slicer; values in cubic millimeters were converted to milliliters, and bilateral volumes were summed to obtain the participant-level total. Midline shift was recorded in millimeters. Internal architecture was classified as homogeneous, laminar, separated or trabecular; a bilateral case with discordant types was reported separately. These imaging descriptors apply only to participants with CSDH and were not coded as zero in controls or entered as common case-control covariates. Postoperative recurrence was not used as a baseline adjustment variable. Medication indicators denoted statin, anticoagulant and antiplatelet use before admission in CSDH cases and current use at the health examination or fecal sampling in controls. CSDH cases with anticoagulant or antiplatelet exposure had discontinued these drugs 7 days before admission. Dietary intake and nutritional status were not systematically assessed.

### Fecal sample collection

We collected fecal samples from all participants using standardized preanalytical procedures intended to limit technical variation in microbiome studies (Goodrich et al., 2014). Fecal samples from participants with CSDH were collected on the morning after hospital admission while they were fasting. Samples were collected before burr-hole drainage and placed in clean containers. Samples were stored at -20 °C, transported at -20 °C within 24 h, and then stored at -80 °C until DNA extraction. We extracted bacterial DNA, amplified the V3-V4 region of the 16S rRNA gene, and sequenced the amplicons on an Illumina NovaSeq platform. The **Supplementary Methods** provide detailed procedures for DNA extraction, amplicon preparation, library construction, and sequencing.

### Microbiome bioinformatics

Paired-end reads were demultiplexed, stripped of barcode and primer sequences, merged with FLASH (Magoč and Salzberg, 2011), quality filtered, and screened for chimeras with VSEARCH (Rognes et al., 2016). High-quality sequences were clustered into operational taxonomic units (OTUs) at 97% similarity using UPARSE (Edgar, 2013), and representative sequences were taxonomically annotated against SILVA release 138.1 (Quast et al., 2013) using QIIME (Caporaso et al., 2010). The 97% OTU approach was the sequencing provider’s default workflow for this project. We retained the resulting OTU feature definition for the additional covariate and read-depth sensitivity analyses to keep their inputs comparable. OTU counts were rarefied to 46,530 sequences per sample, the smallest retained library, so that all 136 participants could be included. The normalized table supported the original diversity and relative-abundance analyses; non-rarefied genus counts were additionally examined in sensitivity analyses. We quantified alpha diversity using the Chao1, Shannon, and Simpson indices (Chao, 1984; Shannon, 1948; Simpson, 1949). We assessed beta diversity using Bray-Curtis (Bray and Curtis, 1957), weighted UniFrac, and unweighted UniFrac distances (Lozupone and Knight, 2005; Lozupone et al., 2007) and visualized it by principal coordinate analysis. We summarized taxonomic composition at the phylum and genus levels. Linear discriminant analysis effect size was used to explore differential taxa (Segata et al., 2011), followed by covariate-adjusted centered log-ratio (CLR) models that accounted for the compositional nature of sequencing data (Gloor et al., 2017). We evaluated genus-level discrimination using a random forest classifier (Breiman, 2001) with nested cross-validation. Partial Spearman correlations tested associations with clinical characteristics. **Figure 1A** summarizes the study design and analytical workflow. The **Supplementary Methods** describe all preprocessing parameters, feature filters, model settings, and correlation procedures.

**Figure 1:**
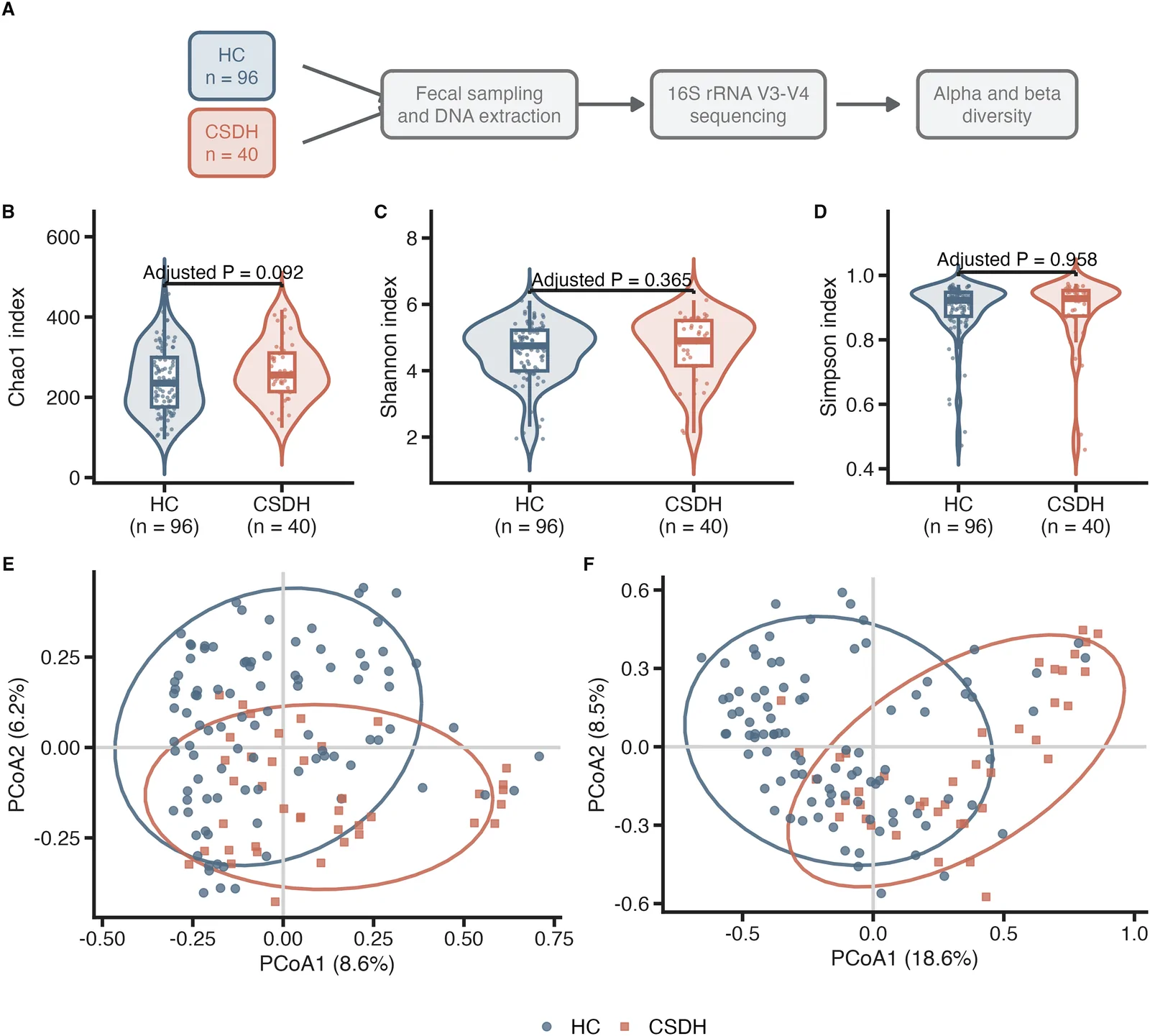
Gut microbial diversity and community structure in participants with chronic subdural hematoma and healthy controls. **(A)** Study design and analytical workflow. Fecal samples from 40 participants with chronic subdural hematoma (CSDH) and 96 healthy controls (HC) were analyzed by 16S rRNA V3–V4 amplicon sequencing. **(B–D)** Chao1 richness **(B)**, Shannon diversity **(C)** and Simpson diversity **(D)**. Each point represents one participant; violin outlines show the distribution, boxes show the median and interquartile range, and whiskers extend to 1.5 times the interquartile range. P values were obtained from two-sided linear models adjusted for age, sex, hypertension, smoking and alcohol use. **(E, F)** Principal-coordinate analysis based on Bray–Curtis **(E)** and weighted UniFrac **(F)** distances. Ellipses indicate 80% normal regions. Group differences were assessed by partial PERMANOVA adjusted for the same covariates with 999 permutations (Bray–Curtis: pseudo-F = 5.029, R² = 0.036, P = 0.001; weighted UniFrac: pseudo-F = 16.297, R² = 0.106, P = 0.001). HC, healthy control; CSDH, chronic subdural hematoma; PCoA, principal-coordinate analysis; PERMANOVA, permutational multivariate analysis of variance.

### Statistical analysis

We summarized continuous baseline variables as medians and interquartile ranges and compared groups using the Wilcoxon rank-sum test. Categorical variables were reported as counts and percentages and compared using Pearson’s chi-square test or Fisher’s exact test, as appropriate. We also calculated absolute standardized mean differences. Linear models adjusted for age, sex, hypertension, current smoking, and alcohol use estimated group differences in alpha diversity. Partial permutational multivariate analysis of variance (PERMANOVA), adjusted for the same covariates, tested beta-diversity differences (Anderson, 2001). PERMDISP tested differences in multivariate dispersion (Anderson, 2006).

Additional genus-level sensitivity analyses added diabetes to the original covariate set and then further included statin, anticoagulant and antiplatelet exposure. We repeated the three models using relative abundances calculated from the original, non-rarefied genus counts. Benjamini-Hochberg correction was applied separately across all eligible genera within each model and preprocessing variant. Exploratory participant bootstrap analyses assessed the internal stability of the 13 primary genus coefficients using 1,000 resamples stratified by disease group. The **Supplementary Methods** give the model specifications, preprocessing and bootstrap limitations.

The Benjamini-Hochberg procedure controlled the false discovery rate in the genus-level differential abundance and clinical correlation analyses (Benjamini and Hochberg, 1995). All tests were two-sided. We considered p < 0.05 nominally significant and q < 0.05 significant after multiple-testing correction. All 136 participants had complete data for the covariates used in the primary and medication-expanded genus models. The original statistical analyses were performed in R v4.2.0. Random forest models used the randomForest package v4.7-1.2.

## Results

### Clinical characteristics

The analysis included 136 participants, comprising 40 adults with CSDH and 96 HCs. Median age was 69.5 years (interquartile range, 61.8-76.0) in the CSDH group and 68.0 years (62.0-73.0) in the HC group (p = 0.475). The CSDH group had higher proportions of men (90.0% vs. 75.0%, p = 0.049) and participants with diabetes (20.0% vs. 7.3%, p = 0.039). It had a lower proportion of current smokers (30.0% vs. 50.0%, p = 0.032). Other baseline comparisons are reported in **Table 1**.

**Table 1:** Population characteristics of participants with CSDH and healthy controls.

Characteristic	CSDH (n = 40)	HC (n = 96)	P value
Age, years	69.5 (61.8-76.0)	68.0 (62.0-73.0)	0.475
Male sex	36 (90.0%)	72 (75.0%)	0.049
Hypertension	22 (55.0%)	48 (50.0%)	0.595
Diabetes mellitus	8 (20.0%)	7 (7.3%)	0.039
Hyperlipidemia	6 (15.0%)	19 (19.8%)	0.511
Coronary heart disease	3 (7.5%)	12 (12.5%)	0.552
Chronic obstructive pulmonary disease	2 (5.0%)	10 (10.4%)	0.508
Cerebral infarction	3 (7.5%)	19 (19.8%)	0.076
Current smoking	12 (30.0%)	48 (50.0%)	0.032
Alcohol use	13 (32.5%)	31 (32.3%)	0.981
Statin exposure	5 (12.5%)	16 (16.7%)	0.540
Anticoagulant exposure	1 (2.5%)	4 (4.2%)	1.000
Antiplatelet exposure	4 (10.0%)	7 (7.3%)	0.731

Participant-level characteristics in the complete analysis cohort. Age is median (IQR); categorical variables are n (%). Sex was coded 0 = female and 1 = male. Age was compared using a two-sided Wilcoxon rank-sum test. Categorical variables were compared using Pearson’s chi-square test or Fisher’s exact test as appropriate. P values are descriptive and unadjusted. CSDH, chronic subdural hematoma; HC, healthy control. Medication variables indicate use before admission in CSDH cases and current use at health examination or fecal sampling in controls. CSDH cases who had used anticoagulants or antiplatelets had stopped these drugs 7 days before admission. The additional medication comparisons used Pearson’s chi-square test for statins and Fisher’s exact test for anticoagulants and antiplatelets.

Statin exposure was recorded in 5/40 participants with CSDH (12.5%) and 16/96 HCs (16.7%; p = 0.540). Anticoagulant exposure was recorded in 1/40 (2.5%) and 4/96 (4.2%; p = 1.000), and antiplatelet exposure in 4/40 (10.0%) and 7/96 (7.3%; p = 0.731), respectively (**Table 1**). These indicators represent pre-admission use in cases and current use at assessment in controls. Among participants with CSDH, median total hematoma volume was 111.77 mL (interquartile range, 82.36-127.53). Median midline shift was 8.77 mm (interquartile range, 4.82-11.23). Internal architecture was homogeneous in 18 participants (45.0%), laminar in 2 (5.0%), separated in 4 (10.0%), and trabecular in 15 (37.5%); one participant (2.5%) had different types on the two sides. One participant (2.5%) had postoperative recurrence within 180 days. These descriptors are provided in **Supplementary Table S7**.

Across all samples, the median number of retained sequences was 86,665 (range, 46,530-139,003), and the median Good’s coverage was 0.999 (range, 0.998-1.000). There was no clear evidence of between-group differences in retained sequence counts (p = 0.621) or Good’s coverage (p = 0.749; **Supplementary Figures S1A, B**). Observed feature and Shannon rarefaction curves approached plateaus by the last plotted depth of 46,528 sequences, close to the analytical subsampling depth of 46,530 sequences per sample (**Supplementary Figures S1C, D**).

### Alpha and beta diversity between the CSDH and HC groups

There was no clear evidence of an alpha-diversity difference between the CSDH and HC groups after adjustment for age, sex, hypertension, current smoking, and alcohol use. The adjusted CSDH-HC coefficient for Chao1 richness was 27.043 (95% CI, -4.515 to 58.602; p = 0.092). The coefficient for Shannon diversity was 0.168 (95% CI, -0.197 to 0.533; p = 0.365). For Simpson diversity, the coefficient was -0.001 (95% CI, -0.040 to 0.038; p = 0.958). These results are shown in **Figures 1B–D** and **Supplementary Table S1**.

Beta diversity differed between the CSDH and HC groups for all three distance measures. Partial PERMANOVA detected a difference in Bray-Curtis distance (pseudo-F = 5.029, R² = 0.036, p = 0.001). Weighted UniFrac distance also differed (pseudo-F = 16.297, R² = 0.106, p = 0.001; **Figures 1E, F**). Unweighted UniFrac distance showed a similar difference (pseudo-F = 4.009, R² = 0.029, p = 0.001). There was no evidence of a between-group dispersion difference for Bray-Curtis (PERMDISP p = 0.951), weighted UniFrac (p = 0.275), or unweighted UniFrac (F = 0.013, p = 0.911; **Supplementary Figures S2A, B**). **Supplementary Table S1** provides the complete adjusted diversity and dispersion results. The adjusted group term accounted for 10.6% of total weighted UniFrac variation.

### Taxonomic alterations between the CSDH and HC groups

Microbial composition varied among participants at the phylum and genus levels and in sample-resolved CLR profiles (**Figures 2A–D**). The mean relative abundances of the predominant phyla are summarized in **Table 2**. Linear discriminant analysis effect size identified 20 discriminatory features across taxonomic ranks. These features met Kruskal-Wallis p < 0.05 and a linear discriminant analysis score >4.0 (**Figures 3A, B**; **Supplementary Figures S3A, B**; **Supplementary Table S2**). At the genus level, *Bacteroides* was enriched in the HC group. *Collinsella*, *Blautia*, and *Escherichia*-*Shigella* were enriched in the CSDH group. After covariate adjustment, *Bacteroides*, *Collinsella*, and *Blautia* remained significant, whereas *Escherichia*-*Shigella* did not (**Supplementary Figure S3C**).

**Figure 2:**
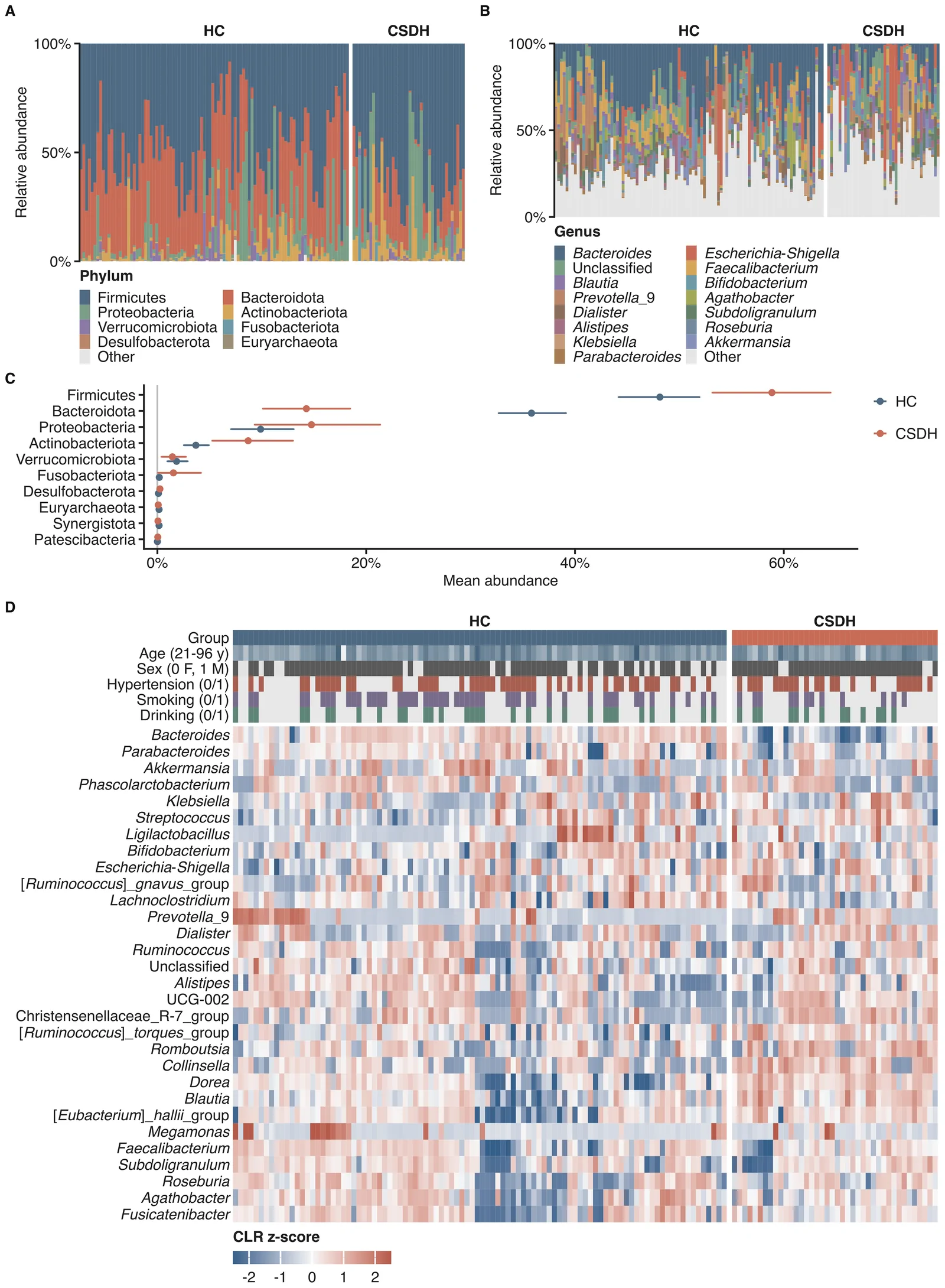
Gut microbial composition in participants with chronic subdural hematoma and healthy controls. **(A)** Sample-level relative abundance of the eight most abundant phyla; the remaining phyla are grouped as Other. **(B)** Sample-level relative abundance of the 15 most abundant genera; the remaining genera are grouped as Other. Vendor-derived “Others” entries are labeled Unclassified. In (A,B), all 136 samples are shown and samples are clustered within each study group by Ward’s method using centered log-ratio (CLR)-transformed profiles of the 30 most abundant genera. **(C)** Mean relative abundance of the 10 most abundant phyla in each group. Points indicate means and horizontal bars indicate nonparametric 95% bootstrap confidence intervals based on 2,000 resamples of participants. **(D)** Sample-resolved heatmap of the 30 most abundant genera. Relative abundances were CLR-transformed using a pseudocount equal to half the smallest non-zero abundance and standardized within each genus. Genera were ordered by Ward hierarchical clustering. Annotation tracks show group, age, sex, hypertension, smoking and drinking status. Heatmap values were truncated at ±2.5 for display. HC, healthy control (n = 96); CSDH, chronic subdural hematoma (n = 40); CLR, centered log-ratio. Sex was coded as 0, female and 1, male; binary clinical variables were coded as 0, absent and 1, present.

**Table 2:** Relative abundance of the predominant bacterial phyla.

Phylum	CSDH (n = 40)	HC (n = 96)	P value	BH q value
Firmicutes	58.86 ± 18.53	48.13 ± 19.06	0.005	0.013
Bacteroidota	14.27 ± 13.38	35.83 ± 16.14	<0.001	<0.001
Proteobacteria	14.76 ± 19.68	9.89 ± 14.81	0.192	0.256
Actinobacteriota	8.70 ± 12.65	3.69 ± 6.00	<0.001	<0.001
Verrucomicrobiota	1.44 ± 3.81	1.84 ± 4.91	0.601	0.686
Fusobacteriota	1.53 ± 7.74	0.18 ± 0.88	0.147	0.235
Desulfobacterota	0.24 ± 0.34	0.10 ± 0.18	0.018	0.035
Euryarchaeota	0.08 ± 0.43	0.16 ± 0.76	0.952	0.952

The eight most abundant phyla across all 136 participants are shown. Values are mean ± s.d. relative abundance (%). Two-sided Wilcoxon rank-sum P values were Benjamini-Hochberg corrected across the eight displayed phyla. BH, Benjamini-Hochberg; CSDH, chronic subdural hematoma; HC, healthy control.

**Figure 3:**
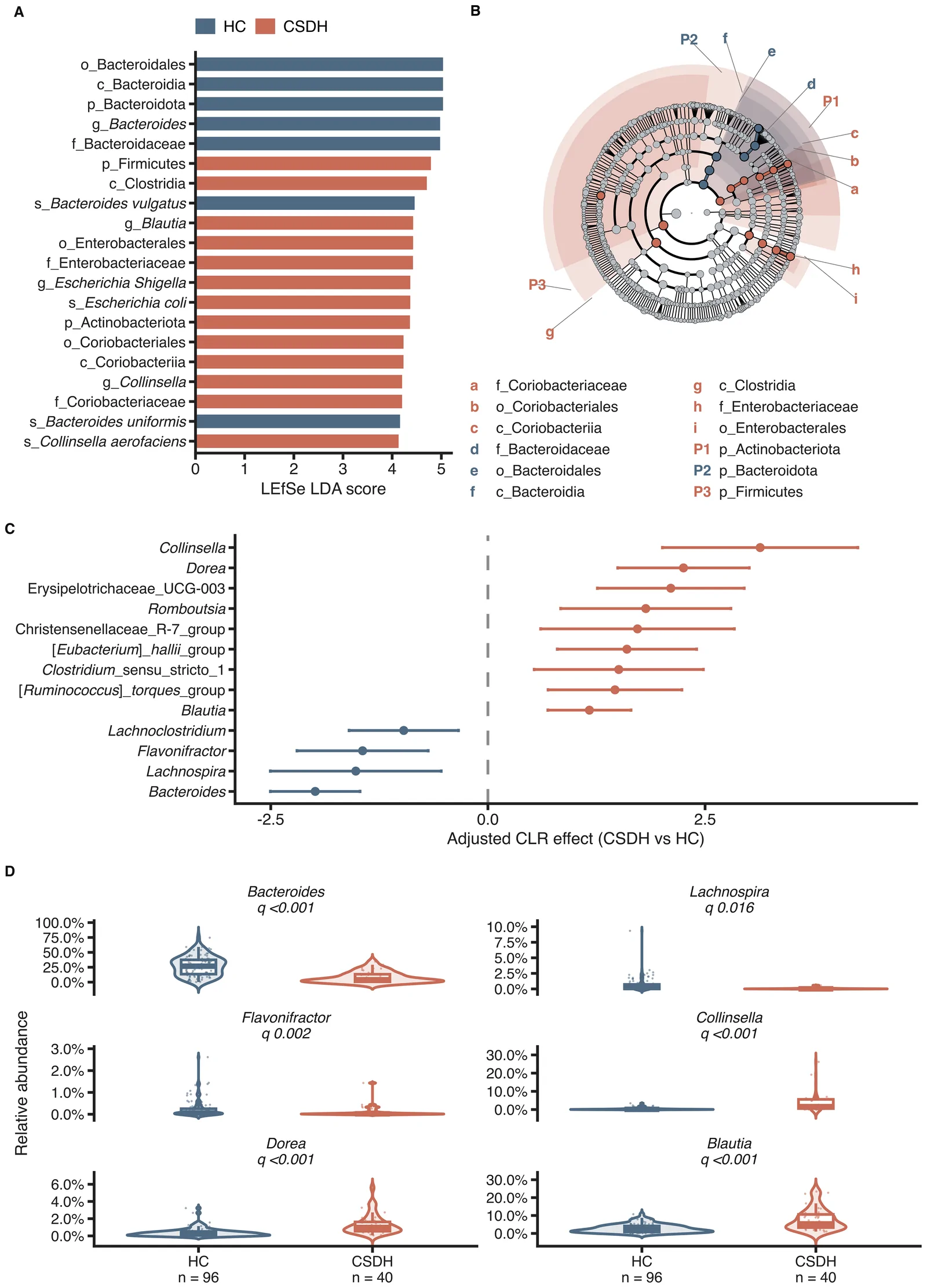
Differential gut microbial taxa between participants with chronic subdural hematoma and healthy controls. **(A)** Linear discriminant analysis effect size (LEfSe) scores for discriminatory taxa from phylum to species. Biomarkers met Kruskal–Wallis P < 0.05 and LDA score > 4.0; colors indicate the group in which each taxon was enriched. **(B)** LEfSe cladogram showing the taxonomic distribution of discriminatory lineages. Colored nodes and sectors denote group-enriched taxa, gray nodes denote non-discriminatory taxa, and node size reflects relative abundance. **(C)** Covariate-adjusted CLR effects and 95% confidence intervals for the 13 genera meeting Benjamini–Hochberg q < 0.05. Genera present in at least 20% of samples and with an overall mean relative abundance of at least 0.1% were retained before modeling (71 of 344 genera). Positive coefficients indicate higher abundance in CSDH and negative coefficients indicate higher abundance in HC. The primary models were adjusted for age, sex, hypertension, smoking and alcohol use. **(D)** Relative-abundance distributions of six representative significant genera. Violin plots show the distributions, boxes show the median and interquartile range, and points represent participants; q values are from the primary adjusted CLR models. Sensitivity analyses with additional diabetes and medication adjustment, non-rarefied counts and exploratory bootstrap resampling are reported in **Supplementary Tables S8**–**S10**, respectively. HC, healthy control (n = 96); CSDH, chronic subdural hematoma (n = 40); CLR, centered log-ratio; LDA, linear discriminant analysis.

Of the 344 detected genera, 71 met the prespecified prevalence and mean-abundance thresholds. Of these, 13 remained significant after covariate adjustment and Benjamini-Hochberg correction. Nine genera were more abundant in the CSDH group: *Dorea, Collinsella, Erysipelotrichaceae UCG-003, Blautia, Eubacterium hallii* group, *Ruminococcus torques* group, *Romboutsia, Clostridium* sensu stricto 1, *and Christensenellaceae R-7* group. Four genera were more abundant in the HC group: *Bacteroides, Flavonifractor, Lachnoclostridium, and Lachnospira* (**Figure 3C**, **Table 3**; **Supplementary Table S3**). *Collinsella* had the largest positive adjusted CLR coefficient (3.134; 95% CI, 2.009-4.259; q < 0.001). *Dorea* had the next largest coefficient (2.251; 95% CI, 1.494-3.009; q < 0.001). *Bacteroides* had the largest negative coefficient (-1.988; 95% CI, -2.504 to -1.472; q < 0.001). **Figure 3D** shows raw abundance distributions for six representative significant genera.

**Table 3:** Covariate-adjusted differential bacterial genera.

Genus	CSDH (n = 40)	HC (n = 96)	Adjusted CLR coefficient (95% CI)	P value	BH q value
*Bacteroides*	8.30 ± 9.29	27.00 ± 16.31	-1.988 (-2.504 to -1.472)	<0.001	<0.001
*Dorea*	1.32 ± 1.17	0.40 ± 0.52	2.251 (1.494 to 3.009)	<0.001	<0.001
*Collinsella*	3.63 ± 5.45	0.35 ± 0.58	3.134 (2.009 to 4.259)	<0.001	<0.001
Erysipelotrichaceae UCG-003	0.63 ± 1.78	0.08 ± 0.15	2.106 (1.260 to 2.951)	<0.001	<0.001
*Blautia*	7.79 ± 6.09	2.80 ± 2.33	1.169 (0.690 to 1.647)	<0.001	<0.001
*Eubacterium hallii* group	1.12 ± 1.19	0.54 ± 0.57	1.600 (0.798 to 2.403)	<0.001	0.002
*Flavonifractor*	0.11 ± 0.24	0.23 ± 0.40	-1.442 (-2.198 to -0.687)	<0.001	0.002
*Ruminococcus torques* group	2.92 ± 3.68	1.17 ± 1.74	1.463 (0.692 to 2.233)	<0.001	0.002
*Romboutsia*	1.60 ± 2.36	0.40 ± 1.23	1.817 (0.838 to 2.797)	<0.001	0.003
*Clostridium* sensu stricto 1	1.20 ± 2.85	0.26 ± 0.65	1.507 (0.533 to 2.480)	0.003	0.016
*Lachnospira*	0.06 ± 0.11	0.64 ± 1.17	-1.521 (-2.505 to -0.537)	0.003	0.016
Christensenellaceae R-7 group	0.87 ± 1.37	0.50 ± 1.60	1.722 (0.607 to 2.837)	0.003	0.016
*Lachnoclostridium*	0.33 ± 0.53	0.72 ± 1.35	-0.969 (-1.599 to -0.339)	0.003	0.016

Only genera passing Benjamini-Hochberg q < 0.05 are shown. Relative abundance values are mean ± s.d. (%). Positive CLR coefficients indicate higher abundance in CSDH; negative coefficients indicate higher abundance in HC. CLR models were adjusted for age, sex, hypertension, smoking and alcohol use. BH correction was applied across 71 tested genera. CLR, centered log-ratio; CI, confidence interval; BH, Benjamini-Hochberg; CSDH, chronic subdural hematoma; HC, healthy control.

All 13 primary associations retained their directions and remained significant after additional adjustment for diabetes, and after further adjustment for statin, anticoagulant and antiplatelet exposure (all q < 0.05; **Supplementary Table S8**). For *Dorea*, the diabetes-adjusted coefficient was 2.054 (95% CI, 1.302-2.807; q < 0.001), compared with 2.251 in the original model; the medication-expanded coefficient was 2.029 (95% CI, 1.268-2.790; q < 0.001). Analyses of non-rarefied counts retained all 13 associations and additionally identified *Olsenella* in each covariate model (**Supplementary Table S9**). In the exploratory bootstrap analysis, pointwise percentile intervals for the original 13 genera excluded zero in all three models (**Supplementary Table S10**).

### Random forest classifier and internal validation

Across 10 repeats of outer cross-validation, the mean out-of-fold area under the curve (AUC) was 0.938 ± 0.010 (range, 0.919-0.947; **Supplementary Figure S4A**; **Supplementary Table S4**). Averaging each participant’s 10 out-of-fold probabilities produced an aggregated AUC of 0.951 (**Figure 4**). The outer models selected a median of 20 genera (interquartile range, 10-30; **Supplementary Figure S4B**). *Bacteroides*, *Lachnospira*, and *Collinsella* were selected in all 50 outer models. *Romboutsia*, *Eubacterium eligens* group, and *Dorea* were selected in 94%, 94%, and 90% of models, respectively (**Supplementary Figure S4C**; **Supplementary Table S5**). These estimates quantify internal discrimination under resampling and do not constitute external validation.

**Figure 4:**
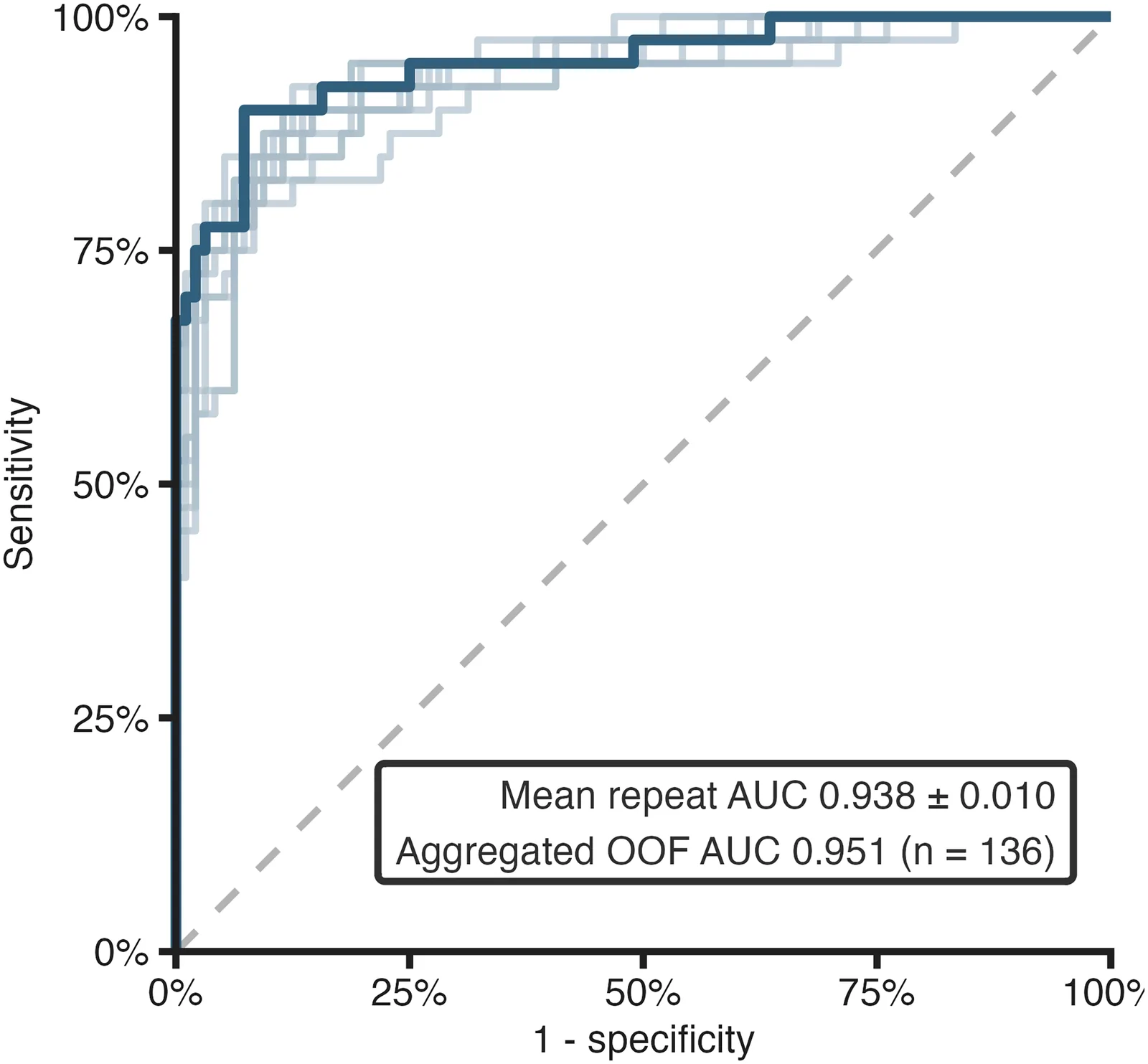
Out-of-fold internal discrimination of the genus-based classifier. Receiver-operating-characteristic curves were generated exclusively from outer-fold predictions for 136 participants (HC, n = 96; CSDH, n = 40) using stratified fivefold outer cross-validation repeated 10 times. Genus filtering, zero replacement, centered log-ratio transformation, random-forest feature ranking and feature-count selection were performed separately within each training fold; inner fivefold cross-validation was used for model selection. Thin curves show the 10 repeat-specific out-of-fold ROC curves, and the thick curve shows the ROC curve obtained after averaging each participant’s 10 out-of-fold probabilities. The mean repeat AUC was 0.938 ± 0.010 (mean ± s.d. across repeated partitions), and the aggregated out-of-fold AUC was 0.951. The s.d. reflects resampling variability and is not a confidence interval. Training-set ROC curves and training-set AUC values are not shown. HC, healthy control; CSDH, chronic subdural hematoma; ROC, receiver operating characteristic; AUC, area under the ROC curve. These curves assess internal ranking discrimination and do not establish calibration or clinical utility.

### Correlation between gut microbiota and clinical characteristics

Of 130 genus-characteristic pairs, two remained significant after adjustment for disease group, age, and sex and correction for multiple testing. *Dorea* abundance was positively correlated with diabetes (partial Spearman r = 0.334; 95% CI, 0.173-0.477; p < 0.001; q = 0.011). *Bacteroides* abundance was negatively correlated with cerebral infarction (r = -0.292; 95% CI, -0.441 to -0.129; p < 0.001; q = 0.041). **Figure 5** and **Supplementary Table S6** provide both associations. The Spearman correlation between adjusted and unadjusted coefficients was 0.818, with 78.5% direction agreement and a median absolute change of 0.039 (**Supplementary Figures S5A, B**). **Supplementary Figure S5C** shows mean absolute coefficients by clinical characteristic.

**Figure 5:**
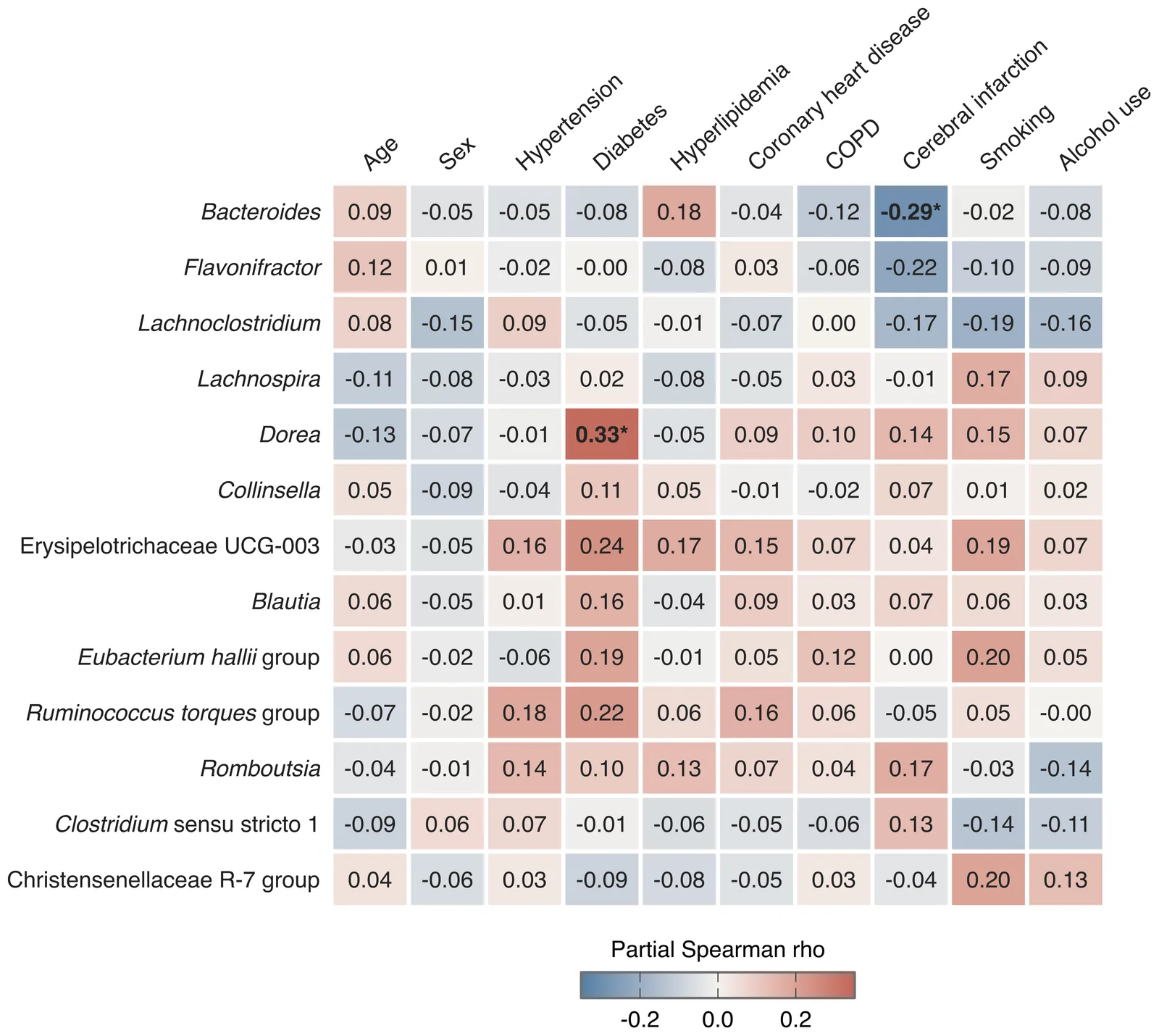
Clinical associations of discriminatory gut genera. Heatmap showing partial Spearman correlations between the 13 genera retained in the adjusted differential-abundance analysis and 10 demographic, vascular and behavioral variables. Genus abundance was centered log-ratio transformed. Disease group was included as a covariate in every analysis; age and sex were additionally adjusted except when the corresponding variable was the target. Cell color and the printed value indicate the partial Spearman correlation coefficient. Benjamini–Hochberg correction was applied across all 130 genus–clinical variable comparisons; *q < 0.05. The analysis included 136 independent participants (HC, n = 96; CSDH, n = 40). Sex was coded as 0, female and 1, male. Associations are cross-sectional and do not establish causality. HC, healthy control; CSDH, chronic subdural hematoma; COPD, chronic obstructive pulmonary disease.

## Discussion

This case-control study found differences in gut microbial composition between adults with CSDH and healthy controls. Community structure differed across three beta-diversity measures, whereas alpha-diversity estimates provided no clear evidence of a group difference. Thirteen genera were associated with CSDH after covariate adjustment and multiple-testing correction. Their directions and statistical significance persisted after additional adjustment for diabetes and recorded medication exposure and in analyses of non-rarefied counts. These results identify exploratory microbial associations in this cohort, without establishing that the microbiota contributes to CSDH development or progression.

Alpha-diversity estimates remain uncertain: the Chao1 interval permits an appreciable increase in richness, the Shannon interval permits differences in either direction, and no biological equivalence margin was defined. For beta diversity, the adjusted group term accounted for 3.6%, 10.6% and 2.9% of total Bray-Curtis, weighted UniFrac and unweighted UniFrac variation, respectively. Most variation was therefore not attributable to disease group. The larger weighted UniFrac estimate is compatible with differences in abundant lineages, although distance-specific effect sizes are not directly interchangeable. PERMDISP found no evidence of unequal dispersion, not proof of identical within-group spread.

Studies of migraine with irritable bowel syndrome, mild cognitive impairment and moyamoya disease provide context, not evidence of CSDH specificity (Liu et al., 2022; Fan et al., 2023; Yu et al., 2023). Differences in recruitment, comorbidity, diet, geography, sample handling and analytical methods prevent direct genus-level comparisons or inference of a shared neurological signature.

Inflammation and angiogenesis are relevant to CSDH biology (Edlmann et al., 2017), but these data do not link microbial associations to either process. Taxonomic profiles from 16S rRNA sequencing do not measure microbial functional genes, metabolic pathways, metabolites, intestinal permeability or circulating immune mediators (Knight et al., 2018). Species and strains within a genus may differ in function, and relative abundance does not measure absolute bacterial load (Gloor et al., 2017). We therefore cannot infer altered metabolite production, inflammatory activity or effects on hematoma membranes from these genus-level findings. Such hypotheses require direct functional measurements and an appropriate longitudinal or experimental design.

The random forest model distinguished the sampled groups under internal resampling, with a mean repeat-level out-of-fold AUC of 0.938 and a secondary aggregated AUC of 0.951. Repeated feature selection indicates consistency within this resampling procedure, not external validity. Nested cross-validation limits feature-selection and tuning bias (Ambroise and McLachlan, 2002; Varma and Simon, 2006), but cannot correct recruitment differences or establish disease specificity. AUC measures ranking discrimination, not calibration or clinical usefulness; the case-control sampling fraction does not reflect clinical prevalence. No calibration analysis or external validation was performed. Independent evaluation of a locked model, calibration and clinical utility is required before diagnostic use can be considered (Collins et al., 2015).

Diabetes adjustment reduced the CSDH coefficient for *Dorea* from 2.251 to 2.054, and further medication adjustment yielded 2.029. Persistence of the association does not exclude residual metabolic or treatment-related confounding. The *Dorea*-diabetes and *Bacteroides*-cerebral infarction correlations were estimated across the full cohort, not specifically among CSDH cases. Shared exposures, reverse causation and differences in medical care remain alternative explanations. Non-rarefied analyses retained the 13 primary genus associations but additionally identified *Olsenella*, indicating broad consistency rather than complete agreement. Rarefaction discards information and may affect low-abundance features.

Recruitment setting was inseparable from disease group: cases were hospitalized, whereas controls attended routine health examinations. Sampling before drainage limits the influence of surgery but does not exclude effects of hospitalization, acute illness, altered intake or medication changes. Differences in general health, frailty and bowel habits could also contribute. Dietary intake and nutritional status were not systematically measured, so differences in habitual diet, recent intake or nutritional condition could contribute to the observed microbial differences and could not be adjusted for. These factors limit disease-specific interpretation of both taxonomic associations and classifier discrimination.

The single-center sample included only 40 participants with CSDH, limiting precision, detection of small effects and generalizability. The medication-expanded models contained ten predictors plus an intercept, with sparse exposure to some drugs. Medication records captured pre-admission use in cases but current use in controls; doses and durations were unavailable, and prior antithrombotic exposure may remain relevant after discontinuation. Adjustment cannot fully account for differences in active or cumulative exposure. The bootstrap intervals assess internal coefficient stability for previously selected genera; they do not establish adequate power or account for feature-selection and multiple-testing uncertainty. Four expanded-model bootstrap samples had rank-deficient designs, illustrating the instability introduced by sparse exposures. Internal hematoma architecture was a clinical classification, not a separate quantitative measure of membrane septations. A single fecal sample cannot establish temporal order or within-person variation. Clustering at 97% similarity can merge distinct amplicon sequences that ASV methods such as DADA2 can resolve (Callahan et al., 2016). Sensitivity analyses using OTU-derived genus tables do not test robustness to ASV inference, and these data cannot establish species- or strain-specific function. Independent longitudinal cohorts are needed to assess temporal and clinical relevance.

## Conclusion

Adults with CSDH showed differences in gut microbial community structure and genus-level composition, without clear evidence of an alpha-diversity difference. The primary genus associations persisted after additional adjustment for diabetes and recorded medication exposure and in non-rarefied sensitivity analyses. These findings remain observational and do not establish microbial mechanisms or diagnostic utility. Multicenter longitudinal studies with detailed dietary, nutritional and medication assessment, direct functional measurements and independent classifier validation are needed to determine their temporal and clinical relevance.

## Data Availability

The raw 16S rRNA gene sequencing data generated in this study have been deposited in the NCBI Sequence Read Archive (SRA) under BioProject accession number PRJNA1509753.
