# Supplementary material for: Altered gut microbiota composition in adults with chronic subdural hematoma: a case-control study
Source: Front Cell Infect Microbiol. 2026 Oct 23;16:1962090. doi: 10.3389/fcimb.2026.1962090 (PMC13739603; doi:10.3389/fcimb.2026.1962090)
Supplement: Supplementary file 1 [file DataSheet1.docx]

Supplementary Material

## 1.1 Bacterial DNA extraction and 16S rRNA sequencing

Total microbial DNA was extracted from fecal samples using the cetyltrimethylammonium bromide method. DNA quality was assessed by 1% agarose gel electrophoresis, and DNA was diluted to 1 ng/μl. The V3-V4 region of the bacterial 16S rRNA gene was amplified using primers CCTAYGGGRBGCASCAG and GGACTACNNGGGTATCTAAT. Thermal cycling comprised an initial denaturation at 98°C for 1 min; 30 cycles of 98°C for 10 s, 50°C for 30 s, and 72°C for 30 s; and a final extension at 72°C for 5 min.

Amplicons were purified, pooled at equimolar concentrations, and used to prepare indexed sequencing libraries. Libraries were quantified by Qubit fluorometry and quantitative PCR, assessed for fragment-size distribution, and sequenced on an Illumina NovaSeq platform to generate 250-bp paired-end reads.

## 1.2 Sequence processing and taxonomic annotation

Paired-end reads were demultiplexed, and barcode and primer sequences were removed. Adapters and reads shorter than 20 bp were removed using cutadapt v3.3. Read pairs were merged with FLASH v1.2.11 using a minimum overlap of 10 bp and a maximum mismatch density of 0.2. Merged reads were quality filtered with fastp v0.23.1 using a quality threshold of Q19 and a maximum low-quality base proportion of 15%.

Chimeric sequences were identified against the SILVA reference database and removed with vsearch v2.16.0. Sequences were clustered into operational taxonomic units (OTUs) at 97% similarity using UPARSE v7.0.1001. Representative sequences were taxonomically annotated against SILVA release 138.1 using the mothur algorithm in QIIME v1.9.1. OTU counts were rarefied to 46,530 sequences per sample, equal to the smallest retained library, retaining all 136 participants. These normalized counts were used for the original diversity and relative-abundance analyses. The rarefaction curves end at 46,528 sequences because this was the final grid point in the plotting procedure; it is not the normalization depth. To assess sensitivity to read subsampling, additional genus models used the original non-rarefied counts. Clustering at 97% similarity followed the sequencing provider's default workflow for this project. The resulting OTU feature definition was retained for the additional covariate and read-depth sensitivity analyses, so these analyses used the same sequence-clustering framework. No ASV-based reanalysis was performed. These sensitivity analyses therefore assess covariate and read-depth choices, not robustness to the sequence-inference method.

## 1.3 Microbial diversity analysis

Alpha diversity was evaluated using the Chao1, Shannon, and Simpson indices. Rarefaction curves and Good's coverage were used to assess sequencing depth. Group differences in alpha-diversity indices were estimated using linear models adjusted for age, sex, hypertension, current smoking, and alcohol use.

Beta diversity was assessed using Bray-Curtis, weighted UniFrac, and unweighted UniFrac distances and was visualized by principal coordinate analysis. Group differences were tested using partial PERMANOVA adjusted for the same covariates with 999 permutations. Homogeneity of multivariate dispersion was assessed using PERMDISP with 999 permutations.

## 1.4 Differential abundance analysis

Relative taxonomic composition was summarized at the phylum and genus levels. Linear discriminant analysis effect size (LEfSe) was used as an exploratory multirank analysis, with features retained at Kruskal-Wallis p < 0.05 and a linear discriminant analysis score >4.0.

Genera were eligible for hypothesis testing if present in at least 20% of the 136 participants and if their overall mean relative abundance was at least 0.1%; 71 of 344 genera met both criteria. A common pseudocount equal to half the smallest positive relative abundance in the complete table (1.0745755 × 10⁻⁵) was added to every entry, including positive values. Natural-log abundances were then centered by their within-participant mean across all 344 genera; the 71-genus filter determined which outcomes were tested, not which genera entered the CLR denominator. For each eligible genus, a linear model estimated the CSDH-HC coefficient adjusted for age, sex, hypertension, current smoking and alcohol use. Conventional t-based 95% confidence intervals and two-sided p values were calculated. The Benjamini-Hochberg procedure was applied across all 71 genus tests within the model.

## 1.5 Random forest model development and internal validation

The internal discriminatory performance of genus-level profiles was evaluated using a random forest classifier with stratified nested cross-validation. The outer loop comprised five folds repeated 10 times, and the inner loop comprised five folds for feature-count selection. Within each training fold, genera were filtered using the prevalence and mean-abundance thresholds described above, zeros were replaced by one-half of the minimum positive training-fold abundance, and retained abundances were centered log-ratio transformed.

Features were ranked by mean decrease in accuracy (permutation importance), rather than mean decrease in Gini impurity, and candidate feature counts of 3, 5, 10, 20, or 30 were compared using inner out-of-fold AUC. Each model contained 500 trees, used the square root of the feature count at each split, and used balanced class sampling to address the unequal group sizes during fitting. All preprocessing, feature ranking, and model selection were confined to the training folds. The principal performance summary was the mean repeat-level AUC from outer out-of-fold probabilities, and each participant's 10 outer predictions were averaged to construct the aggregated out-of-fold receiver operating characteristic curve. No calibration analysis or external validation was performed.

## 1.6 Correlation analysis

Associations between the 13 genera identified in the adjusted differential abundance analysis and 10 demographic, vascular, and behavioral characteristics were evaluated using partial Spearman correlations based on residualized ranks. Disease group was included as an adjustment variable in every analysis. Age and sex were additionally adjusted for except when the corresponding variable was the target characteristic.

Ninety-five percent confidence intervals were calculated using Fisher's z transformation. The Benjamini-Hochberg procedure was applied across all 130 genus-characteristic tests, and q < 0.05 was considered statistically significant.

## 1.7 Sensitivity analyses

Sensitivity analyses first added diabetes to the original CLR model and then included diabetes, statin, anticoagulant and antiplatelet exposure together. The original, diabetes-adjusted and medication-expanded models included 7, 8 and 11 parameters including the intercept (residual degrees of freedom 129, 128 and 125), respectively. All models used the same 136 participants and had full-rank design matrices. Case-only imaging variables were not included as common covariates. For the non-rarefied analysis, each genus count was divided by the participant's total genus count before applying the same pseudocount rule and 344-genus CLR transformation. The prevalence and mean-abundance filters were reapplied, yielding 71 eligible genera. Benjamini-Hochberg correction was performed separately within each of the six combinations of covariate model and preprocessing variant. All estimates are provided in Supplementary Tables S8-S9 and the accompanying analysis file CLR_all_genera_all_models.csv.

Exploratory coefficient-stability analyses used 1,000 bootstrap attempts, resampling 40 CSDH participants and 96 controls with replacement within their respective groups. The same resampling indices were used for the three covariate models. The original 13 significant genera and the original 344-genus CLR transformation were held fixed. Pointwise percentile 95% bootstrap intervals and the proportion of coefficients retaining their original direction are reported. Rank-deficient bootstrap designs were excluded, giving 1,000 valid replicates for the original and diabetes-adjusted models and 996 for the medication-expanded model. These intervals are conditional on the selected genera, do not account for selection or multiplicity, and assess internal stability rather than prospective statistical power or external validity (Supplementary Table S10).

# 2 Supplementary figures

**Figure S1. Sequencing depth and rarefaction analysis.** (A) Retained sequences per participant in healthy controls (HC, n = 96) and participants with chronic subdural hematoma (CSDH, n = 40). (B) Endpoint Good’s coverage. In (A,B), violins show the distribution, boxes show the median and interquartile range, and symbols denote independent participants; displayed P values are from two-sided Wilcoxon rank-sum tests. (C) Observed-feature rarefaction curves. (D) Shannon-diversity rarefaction curves. In (C,D), faint curves denote individual participants, and thick lines and shaded bands denote group means and 95% confidence intervals, respectively. The curves end at 46,528 sequences, the final plotting grid point; the analytical rarefaction depth was 46,530 sequences per sample. HC, healthy control; CSDH, chronic subdural hematoma.

**Figure S2. Unweighted UniFrac community structure and multivariate dispersion.** (A) Principal-coordinate analysis of unweighted UniFrac distances for healthy controls (HC, n = 96) and participants with chronic subdural hematoma (CSDH, n = 40). Symbols denote independent participants and ellipses indicate 95% normal regions. The marginal group effect was estimated by partial PERMANOVA adjusted for age, sex, hypertension, smoking and alcohol use (pseudo-F = 4.009, R² = 0.029, P = 0.001; 999 permutations). (B) Participant distances to the group spatial median. Violins show the distribution, boxes show the median and interquartile range, and symbols denote participants. There was no evidence of a difference in unweighted UniFrac dispersion between groups (PERMDISP F = 0.013, P = 0.911; 999 permutations). PCoA, principal-coordinate analysis; PERMANOVA, permutational multivariate analysis of variance; PERMDISP, permutational analysis of multivariate dispersions.

**Figure S3. Multirank LEfSe features and genus-level robustness.** (A) Signed linear discriminant analysis (LDA) scores for all 20 discriminatory taxonomic features identified by LEfSe (Kruskal–Wallis P < 0.05 and LDA score > 4.0). Negative and positive values indicate enrichment in healthy controls (HC) and chronic subdural hematoma (CSDH), respectively; prefixes indicate phylum (p), class (C), order (o), family (F), genus (g) and species (s). (B) Numbers of HC- and CSDH-enriched features at each taxonomic rank. (C) Covariate-adjusted centered log-ratio effects and 95% confidence intervals for the four LEfSe-positive genera. The primary models compare CSDH with HC and were adjusted for age, sex, hypertension, smoking and alcohol use; solid symbols indicate Benjamini–Hochberg q < 0.05 across 71 tested genera. Additional covariate and read-depth sensitivity analyses are reported in Supplementary Tables S8 and S9, respectively. LEfSe, linear discriminant analysis effect size.

**Figure S4. Internal stability of the genus-based classifier.** (A) Repeat-level out-of-fold area under the receiver-operating-characteristic curve (AUC) from stratified fivefold outer cross-validation repeated 10 times. The dashed line indicates the mean AUC and the shaded band indicates mean ± s.d. across repeats. The s.d. reflects variation among repeated partitions and is not a confidence interval. (B) Number of genera selected in each of the 50 outer training models. Candidate feature counts of 3, 5, 10, 20 or 30 were compared by stratified fivefold inner cross-validation; numbers above bars denote the numbers of outer models. (C) The 20 genera with the highest selection frequencies across the 50 outer models. Labels show the number of selections out of 50; terracotta points indicate genera selected in at least 80% of outer models. Feature preprocessing, ranking and model selection were repeated within each outer training set. Training-set ROC/AUC and exploratory internal-test AUC are not shown. CV, cross-validation.

**Figure S5. Sensitivity analysis of clinical associations.** (A) Concordance between unadjusted Spearman correlation coefficients and covariate-adjusted partial Spearman coefficients for all 130 combinations of 13 discriminatory genera and 10 prespecified clinical variables. The dashed line indicates equality, and terracotta points indicate the two associations meeting Benjamini–Hochberg q < 0.05 after adjustment. Concordance across paired coefficients was 0.82, 78.5% retained the same direction, and the median absolute change was 0.039. (B) Unadjusted and adjusted correlation coefficients with Fisher z-transformed 95% confidence intervals for the two associations meeting the adjusted false-discovery-rate threshold. For Dorea and diabetes, the unadjusted rho was 0.321 (95% CI, 0.161–0.464; q = 0.018) and the adjusted partial rho was 0.334 (95% CI, 0.173–0.477; q = 0.011). For Bacteroides and cerebral infarction, the unadjusted rho was −0.113 (95% CI, −0.276 to 0.056; q = 0.899) and the adjusted partial rho was −0.292 (95% CI, −0.441 to −0.129; q = 0.041). (C) Mean absolute correlation coefficient across the 13 genera before and after adjustment, summarized by clinical variable. Panel (C) is descriptive and no between-variable hypothesis test was performed. Adjusted partial Spearman correlations were calculated by residualizing ranked variables. Disease group was included in every adjusted analysis; age and sex were additionally adjusted except when the corresponding variable was the target. Benjamini–Hochberg correction was applied separately across the 130 unadjusted and 130 adjusted tests. Genus abundance was centered log-ratio transformed. The analysis included 136 independent participants (HC, n = 96; CSDH, n = 40). Sex was coded as 0, female and 1, male.

# 3 Supplementary tables

**Table S1. Complete alpha- and beta-diversity statistics** Adjusted alpha-diversity models, partial PERMANOVA, and PERMDISP results. For alpha diversity, effects represent the adjusted CSDH-minus-HC difference. Partial PERMANOVA models adjust for age, sex, hypertension, smoking, and alcohol use. PERMDISP tests assess between-group dispersion. CI, confidence interval.

| **Analysis** | **Metric** | **Effect (95% CI)** | **Statistic** | **R²** | **P value** | **Permutations** | **Adjustment** |
| --- | --- | --- | --- | --- | --- | --- | --- |
| Adjusted linear model | Chao1 richness | 27.043 (-4.515, 58.602) | t = 1.695 |  | 0.092 |  | Age; sex; hypertension; smoking; alcohol use |
| Adjusted linear model | Shannon diversity | 0.168 (-0.197, 0.533) | t = 0.908 |  | 0.365 |  | Age; sex; hypertension; smoking; alcohol use |
| Adjusted linear model | Simpson diversity | -0.001 (-0.040, 0.038) | t = -0.052 |  | 0.958 |  | Age; sex; hypertension; smoking; alcohol use |
| Partial PERMANOVA | Bray-Curtis |  | pseudo-F = 5.029 | 0.036 | 0.001 | 999 | Age; sex; hypertension; smoking; alcohol use |
| Partial PERMANOVA | Weighted UniFrac |  | pseudo-F = 16.297 | 0.106 | 0.001 | 999 | Age; sex; hypertension; smoking; alcohol use |
| Partial PERMANOVA | Unweighted UniFrac |  | pseudo-F = 4.009 | 0.029 | 0.001 | 999 | Age; sex; hypertension; smoking; alcohol use |
| PERMDISP | Bray-Curtis |  | F = 0.003 |  | 0.951 | 999 | Not applicable |
| PERMDISP | Weighted UniFrac |  | F = 1.280 |  | 0.275 | 999 | Not applicable |
| PERMDISP | Unweighted UniFrac |  | F = 0.013 |  | 0.911 | 999 | Not applicable |

**Table S2. Complete multirank LEfSe biomarker list** All taxa meeting the prespecified Kruskal-Wallis and LDA thresholds. LEfSe biomarkers satisfy Kruskal-Wallis P < 0.05 and LDA score > 4.0. P values are unadjusted and the analysis should be interpreted as exploratory. LDA, linear discriminant analysis.

| **Rank** | **Taxon** | **Enriched group** | **LDA score** | **P value** | **Mean log10 abundance** | **Full taxonomy** |
| --- | --- | --- | --- | --- | --- | --- |
| Order | Bacteroidales | HC | 5.034 | 2.81e-10 | 5.554 | k__Bacteria.p__Bacteroidota.c__Bacteroidia.o__Bacteroidales |
| Class | Bacteroidia | HC | 5.034 | 2.81e-10 | 5.554 | k__Bacteria.p__Bacteroidota.c__Bacteroidia |
| Phylum | Bacteroidota | HC | 5.034 | 2.81e-10 | 5.554 | k__Bacteria.p__Bacteroidota |
| Family | Bacteroidaceae | HC | 4.975 | 2.48e-10 | 5.431 | k__Bacteria.p__Bacteroidota.c__Bacteroidia.o__Bacteroidales.f__Bacteroidaceae |
| Genus | Bacteroides | HC | 4.975 | 2.48e-10 | 5.431 | k__Bacteria.p__Bacteroidota.c__Bacteroidia.o__Bacteroidales.f__Bacteroidaceae.g__Bacteroides |
| Phylum | Firmicutes | CSDH | 4.788 | 0.005 | 5.770 | k__Bacteria.p__Firmicutes |
| Class | Clostridia | CSDH | 4.705 | 0.004 | 5.672 | k__Bacteria.p__Firmicutes.c__Clostridia |
| Species | Bacteroides vulgatus | HC | 4.457 | 4.19e-06 | 4.874 | k__Bacteria.p__Bacteroidota.c__Bacteroidia.o__Bacteroidales.f__Bacteroidaceae.g__Bacteroides.s__Bacteroides_vulgatus |
| Genus | Blautia | CSDH | 4.427 | 8.61e-08 | 4.891 | k__Bacteria.p__Firmicutes.c__Clostridia.o__Lachnospirales.f__Lachnospiraceae.g__Blautia |
| Order | Enterobacterales | CSDH | 4.426 | 0.021 | 5.146 | k__Bacteria.p__Proteobacteria.c__Gammaproteobacteria.o__Enterobacterales |
| Family | Enterobacteriaceae | CSDH | 4.424 | 0.018 | 5.136 | k__Bacteria.p__Proteobacteria.c__Gammaproteobacteria.o__Enterobacterales.f__Enterobacteriaceae |
| Genus | Escherichia Shigella | CSDH | 4.369 | 0.045 | 5.033 | k__Bacteria.p__Proteobacteria.c__Gammaproteobacteria.o__Enterobacterales.f__Enterobacteriaceae.g__Escherichia_Shigella |
| Species | Escherichia coli | CSDH | 4.369 | 0.045 | 5.033 | k__Bacteria.p__Proteobacteria.c__Gammaproteobacteria.o__Enterobacterales.f__Enterobacteriaceae.g__Escherichia_Shigella.s__Escherichia_coli |
| Phylum | Actinobacteriota | CSDH | 4.361 | 1.30e-04 | 4.939 | k__Bacteria.p__Actinobacteriota |
| Class | Coriobacteriia | CSDH | 4.231 | 3.17e-10 | 4.622 | k__Bacteria.p__Actinobacteriota.c__Coriobacteriia |
| Order | Coriobacteriales | CSDH | 4.231 | 3.17e-10 | 4.622 | k__Bacteria.p__Actinobacteriota.c__Coriobacteriia.o__Coriobacteriales |
| Genus | Collinsella | CSDH | 4.202 | 4.55e-09 | 4.560 | k__Bacteria.p__Actinobacteriota.c__Coriobacteriia.o__Coriobacteriales.f__Coriobacteriaceae.g__Collinsella |
| Family | Coriobacteriaceae | CSDH | 4.202 | 4.69e-09 | 4.560 | k__Bacteria.p__Actinobacteriota.c__Coriobacteriia.o__Coriobacteriales.f__Coriobacteriaceae |
| Species | Bacteroides uniformis | HC | 4.160 | 5.64e-04 | 4.676 | k__Bacteria.p__Bacteroidota.c__Bacteroidia.o__Bacteroidales.f__Bacteroidaceae.g__Bacteroides.s__Bacteroides_uniformis |
| Species | Collinsella aerofaciens | CSDH | 4.131 | 2.26e-08 | 4.509 | k__Bacteria.p__Actinobacteriota.c__Coriobacteriia.o__Coriobacteriales.f__Coriobacteriaceae.g__Collinsella.s__Collinsella_aerofaciens |

**Table S3. All covariate-adjusted genus-level models** All 71 genera passing prevalence and mean-abundance filters. Positive coefficients indicate higher CLR-transformed abundance in CSDH and negative coefficients indicate higher abundance in HC. Models adjust for age, sex, hypertension, smoking, and alcohol use. BH correction was applied across all 71 tested genera. CLR, centered log-ratio; SE, standard error; BH, Benjamini-Hochberg.

| **Genus** | **Adjusted CLR coefficient (95% CI)** | **SE** | **P value** | **BH q value** | **Prevalence** | **Mean abundance** | **Direction** |
| --- | --- | --- | --- | --- | --- | --- | --- |
| Bacteroides | -1.988 (-2.504, -1.472) | 0.261 | 4.83e-12 | 3.43e-10 | 100.0% | 21.5% | Higher in HC |
| Dorea | 2.251 (1.494, 3.009) | 0.383 | 3.29e-08 | 1.17e-06 | 90.4% | 0.7% | Higher in CSDH |
| Collinsella | 3.134 (2.009, 4.259) | 0.569 | 1.86e-07 | 4.41e-06 | 71.3% | 1.3% | Higher in CSDH |
| Erysipelotrichaceae UCG-003 | 2.106 (1.260, 2.951) | 0.427 | 2.50e-06 | 4.44e-05 | 73.5% | 0.2% | Higher in CSDH |
| Blautia | 1.169 (0.690, 1.647) | 0.242 | 3.82e-06 | 5.42e-05 | 100.0% | 4.3% | Higher in CSDH |
| Eubacterium hallii group | 1.600 (0.798, 2.403) | 0.405 | 1.30e-04 | 0.002 | 90.4% | 0.7% | Higher in CSDH |
| Flavonifractor | -1.442 (-2.198, -0.687) | 0.382 | 2.40e-04 | 0.002 | 89.7% | 0.2% | Higher in HC |
| Ruminococcus torques group | 1.463 (0.692, 2.233) | 0.389 | 2.60e-04 | 0.002 | 96.3% | 1.7% | Higher in CSDH |
| Romboutsia | 1.817 (0.838, 2.797) | 0.495 | 3.53e-04 | 0.003 | 81.6% | 0.7% | Higher in CSDH |
| Clostridium sensu stricto 1 | 1.507 (0.533, 2.480) | 0.492 | 0.003 | 0.016 | 72.8% | 0.5% | Higher in CSDH |
| Lachnospira | -1.521 (-2.505, -0.537) | 0.497 | 0.003 | 0.016 | 77.9% | 0.5% | Higher in HC |
| Christensenellaceae R-7 group | 1.722 (0.607, 2.837) | 0.564 | 0.003 | 0.016 | 64.7% | 0.6% | Higher in CSDH |
| Lachnoclostridium | -0.969 (-1.599, -0.339) | 0.318 | 0.003 | 0.016 | 97.8% | 0.6% | Higher in HC |
| Olsenella | 0.912 (0.187, 1.638) | 0.367 | 0.014 | 0.072 | 40.4% | 0.2% | Not significant |
| Coprococcus | 1.007 (0.159, 1.854) | 0.428 | 0.020 | 0.096 | 89.7% | 0.6% | Not significant |
| Streptococcus | 0.914 (0.078, 1.751) | 0.423 | 0.032 | 0.144 | 97.1% | 1.1% | Not significant |
| Monoglobus | 0.754 (0.003, 1.504) | 0.379 | 0.049 | 0.205 | 84.6% | 0.1% | Not significant |
| Sutterella | -0.916 (-1.843, 0.011) | 0.469 | 0.053 | 0.209 | 61.8% | 0.2% | Not significant |
| Ruminococcus | 1.039 (-0.029, 2.106) | 0.540 | 0.056 | 0.211 | 86.0% | 1.5% | Not significant |
| Intestinibacter | 0.867 (-0.036, 1.769) | 0.456 | 0.060 | 0.212 | 83.8% | 0.3% | Not significant |
| Lactonifactor | 0.710 (-0.042, 1.461) | 0.380 | 0.064 | 0.216 | 43.4% | 0.1% | Not significant |
| UCG-002 | 1.059 (-0.185, 2.302) | 0.629 | 0.095 | 0.295 | 75.7% | 1.7% | Not significant |
| Parasutterella | -0.936 (-2.046, 0.174) | 0.561 | 0.098 | 0.295 | 62.5% | 0.4% | Not significant |
| Enterococcus | -0.865 (-1.907, 0.177) | 0.526 | 0.103 | 0.295 | 57.4% | 0.6% | Not significant |
| Lachnospiraceae ND3007 group | -0.687 (-1.516, 0.143) | 0.419 | 0.104 | 0.295 | 75.7% | 0.1% | Not significant |
| Holdemanella | 0.753 (-0.199, 1.704) | 0.481 | 0.120 | 0.327 | 23.5% | 0.2% | Not significant |
| Veillonella | -0.797 (-1.843, 0.248) | 0.529 | 0.134 | 0.344 | 77.9% | 0.5% | Not significant |
| UCG-005 | 0.802 (-0.261, 1.865) | 0.537 | 0.138 | 0.344 | 75.0% | 0.6% | Not significant |
| Fusicatenibacter | 0.671 (-0.243, 1.584) | 0.462 | 0.149 | 0.344 | 91.2% | 0.9% | Not significant |
| Megasphaera | -0.749 (-1.770, 0.272) | 0.516 | 0.149 | 0.344 | 36.8% | 0.3% | Not significant |
| Dialister | 0.989 (-0.363, 2.340) | 0.683 | 0.150 | 0.344 | 77.9% | 2.6% | Not significant |
| Anaerostipes | 0.504 (-0.218, 1.226) | 0.365 | 0.170 | 0.376 | 95.6% | 0.4% | Not significant |
| Escherichia-Shigella | 0.725 (-0.351, 1.802) | 0.544 | 0.185 | 0.398 | 98.5% | 7.4% | Not significant |
| UBA1819 | -0.474 (-1.233, 0.284) | 0.383 | 0.218 | 0.456 | 89.7% | 0.4% | Not significant |
| NK4A214 group | 0.687 (-0.428, 1.802) | 0.563 | 0.225 | 0.456 | 65.4% | 0.4% | Not significant |
| Sellimonas | -0.543 (-1.464, 0.379) | 0.466 | 0.246 | 0.483 | 48.5% | 0.1% | Not significant |
| CAG-56 | 0.598 (-0.430, 1.626) | 0.519 | 0.252 | 0.483 | 67.6% | 0.2% | Not significant |
| Roseburia | 0.621 (-0.489, 1.730) | 0.561 | 0.270 | 0.503 | 89.0% | 2.0% | Not significant |
| Parabacteroides | -0.438 (-1.236, 0.360) | 0.403 | 0.279 | 0.503 | 94.9% | 1.7% | Not significant |
| Lactobacillus | 0.446 (-0.377, 1.269) | 0.416 | 0.286 | 0.503 | 37.5% | 0.1% | Not significant |
| Fusobacterium | 0.500 (-0.432, 1.431) | 0.471 | 0.290 | 0.503 | 37.5% | 0.5% | Not significant |
| Eubacterium eligens group | -0.556 (-1.624, 0.512) | 0.540 | 0.305 | 0.515 | 69.9% | 0.5% | Not significant |
| UCG-003 | 0.371 (-0.479, 1.222) | 0.430 | 0.389 | 0.643 | 69.1% | 0.1% | Not significant |
| Akkermansia | -0.573 (-1.938, 0.792) | 0.690 | 0.408 | 0.658 | 59.6% | 1.7% | Not significant |
| Megamonas | -0.459 (-1.607, 0.689) | 0.580 | 0.430 | 0.671 | 27.2% | 1.5% | Not significant |
| Bifidobacterium | 0.406 (-0.620, 1.433) | 0.519 | 0.435 | 0.671 | 96.3% | 3.3% | Not significant |
| Clostridium innocuum group | -0.309 (-1.119, 0.500) | 0.409 | 0.451 | 0.681 | 71.3% | 0.1% | Not significant |
| Phascolarctobacterium | -0.457 (-1.716, 0.803) | 0.637 | 0.474 | 0.693 | 73.5% | 1.3% | Not significant |
| Prevotella 9 | 0.517 (-0.922, 1.957) | 0.727 | 0.478 | 0.693 | 37.5% | 3.0% | Not significant |
| Agathobacter | 0.341 (-0.758, 1.440) | 0.556 | 0.541 | 0.768 | 91.2% | 2.6% | Not significant |
| Haemophilus | -0.203 (-1.100, 0.694) | 0.453 | 0.655 | 0.887 | 50.0% | 0.2% | Not significant |
| Citrobacter | 0.202 (-0.727, 1.130) | 0.469 | 0.668 | 0.887 | 54.4% | 0.3% | Not significant |
| Enterobacter | 0.134 (-0.497, 0.764) | 0.319 | 0.676 | 0.887 | 35.3% | 0.2% | Not significant |
| Eubacterium ventriosum group | 0.169 (-0.735, 1.073) | 0.457 | 0.712 | 0.887 | 83.1% | 0.3% | Not significant |
| Alistipes | -0.204 (-1.328, 0.921) | 0.568 | 0.721 | 0.887 | 84.6% | 2.1% | Not significant |
| Eubacterium ruminantium group | -0.184 (-1.228, 0.860) | 0.528 | 0.727 | 0.887 | 33.1% | 0.3% | Not significant |
| Barnesiella | 0.211 (-0.989, 1.410) | 0.606 | 0.729 | 0.887 | 40.4% | 0.4% | Not significant |
| Lachnospiraceae NK4A136 group | 0.155 (-0.792, 1.102) | 0.479 | 0.746 | 0.887 | 80.1% | 0.4% | Not significant |
| Ligilactobacillus | 0.156 (-0.926, 1.238) | 0.547 | 0.776 | 0.887 | 41.9% | 0.7% | Not significant |
| Butyricicoccus | 0.087 (-0.533, 0.707) | 0.313 | 0.781 | 0.887 | 92.6% | 0.1% | Not significant |
| Raoultella | 0.130 (-0.799, 1.059) | 0.469 | 0.782 | 0.887 | 63.2% | 0.2% | Not significant |
| Oscillibacter | -0.114 (-0.938, 0.710) | 0.416 | 0.784 | 0.887 | 84.6% | 0.2% | Not significant |
| Others | 0.056 (-0.357, 0.468) | 0.208 | 0.789 | 0.887 | 100.0% | 6.0% | Not significant |
| Klebsiella | 0.149 (-1.083, 1.381) | 0.623 | 0.811 | 0.887 | 75.7% | 1.9% | Not significant |
| Ruminococcus gnavus group | 0.137 (-0.998, 1.272) | 0.574 | 0.812 | 0.887 | 78.7% | 0.7% | Not significant |
| Butyricimonas | -0.089 (-1.110, 0.933) | 0.516 | 0.864 | 0.929 | 57.4% | 0.3% | Not significant |
| Fecalibacterium | -0.062 (-1.076, 0.951) | 0.512 | 0.903 | 0.957 | 94.9% | 5.8% | Not significant |
| Subdoligranulum | -0.043 (-1.108, 1.021) | 0.538 | 0.936 | 0.968 | 89.0% | 2.5% | Not significant |
| Acinetobacter | 0.024 (-0.636, 0.684) | 0.334 | 0.943 | 0.968 | 22.8% | 0.2% | Not significant |
| Limosilactobacillus | -0.022 (-1.035, 0.991) | 0.512 | 0.966 | 0.968 | 47.8% | 0.4% | Not significant |
| Erysipelatoclostridium | 0.015 (-0.754, 0.784) | 0.389 | 0.968 | 0.968 | 64.0% | 0.1% | Not significant |

**Table S4. Repeat-level nested cross-validation performance** Outer-fold results from 10 repeated stratified five-fold cross-validation runs. Feature-count selection was performed only within the inner five-fold cross-validation loop. AUC values therefore represent outer-fold predictions and are not training-set performance. AUC, area under the receiver operating characteristic curve. Mean selected genera is the participant-weighted mean of the feature counts used by the five outer models, with weights equal to the numbers of held-out participants.

| **Outer repeat** | **Participants** | **HC** | **CSDH** | **Out-of-fold AUC** | **Mean selected genera** |
| --- | --- | --- | --- | --- | --- |
| 1 | 136 | 96 | 40 | 0.925 | 21.91 |
| 2 | 136 | 96 | 40 | 0.945 | 14.04 |
| 3 | 136 | 96 | 40 | 0.936 | 15.04 |
| 4 | 136 | 96 | 40 | 0.928 | 25.96 |
| 5 | 136 | 96 | 40 | 0.919 | 15.56 |
| 6 | 136 | 96 | 40 | 0.946 | 22.06 |
| 7 | 136 | 96 | 40 | 0.939 | 11.66 |
| 8 | 136 | 96 | 40 | 0.943 | 18.01 |
| 9 | 136 | 96 | 40 | 0.947 | 28.01 |
| 10 | 136 | 96 | 40 | 0.945 | 15.96 |

**Table S5. Classifier feature-selection stability** Selection frequency across all 50 outer training models. Selection frequency is the number of outer training models containing a genus divided by 50. Mean rank and mean importance are calculated among models in which the genus was selected.

| **Genus** | **Selected outer models** | **Selection frequency** | **Mean rank when selected** | **Mean ranking importance** |
| --- | --- | --- | --- | --- |
| Bacteroides | 50 | 100.0% | 1.10 | 0.0253 |
| Lachnospira | 50 | 100.0% | 2.22 | 0.0154 |
| Collinsella | 50 | 100.0% | 3.88 | 0.0094 |
| Romboutsia | 47 | 94.0% | 6.47 | 0.0058 |
| Eubacterium eligens group | 47 | 94.0% | 6.49 | 0.0055 |
| Dorea | 45 | 90.0% | 5.42 | 0.0068 |
| Flavonifractor | 39 | 78.0% | 10.00 | 0.0037 |
| Erysipelotrichaceae UCG-003 | 38 | 76.0% | 8.97 | 0.0040 |
| Eubacterium hallii group | 36 | 72.0% | 11.56 | 0.0031 |
| Fusicatenibacter | 36 | 72.0% | 12.22 | 0.0030 |
| Lachnospiraceae ND3007 group | 32 | 64.0% | 10.47 | 0.0036 |
| Ruminococcus torques group | 32 | 64.0% | 11.34 | 0.0033 |
| Lachnoclostridium | 32 | 64.0% | 12.72 | 0.0029 |
| Blautia | 29 | 58.0% | 12.93 | 0.0027 |
| Olsenella | 26 | 52.0% | 14.62 | 0.0025 |
| Fecalibacterium | 23 | 46.0% | 15.61 | 0.0024 |
| Parabacteroides | 21 | 42.0% | 18.19 | 0.0018 |
| Streptococcus | 20 | 40.0% | 16.45 | 0.0020 |
| Enterococcus | 20 | 40.0% | 18.60 | 0.0015 |
| Parasutterella | 20 | 40.0% | 18.60 | 0.0017 |
| Turicibacter | 19 | 38.0% | 2.95 | 0.0117 |
| Clostridium sensu stricto 1 | 19 | 38.0% | 16.79 | 0.0020 |
| Sutterella | 13 | 26.0% | 17.38 | 0.0018 |
| Prevotella 9 | 13 | 26.0% | 21.62 | 0.0013 |
| Eubacterium ventriosum group | 12 | 24.0% | 22.92 | 0.0014 |
| Megasphaera | 11 | 22.0% | 19.73 | 0.0014 |
| Agathobacter | 9 | 18.0% | 19.78 | 0.0014 |
| Sellimonas | 8 | 16.0% | 19.12 | 0.0016 |
| Roseburia | 8 | 16.0% | 20.12 | 0.0013 |
| Alistipes | 8 | 16.0% | 21.62 | 0.0013 |
| Lactonifactor | 8 | 16.0% | 21.75 | 0.0013 |
| Eubacterium ruminantium group | 8 | 16.0% | 22.25 | 0.0012 |
| Intestinibacter | 7 | 14.0% | 19.29 | 0.0016 |
| Holdemanella | 7 | 14.0% | 23.86 | 0.0012 |
| Acinetobacter | 5 | 10.0% | 20.40 | 0.0014 |
| CAG-56 | 5 | 10.0% | 20.40 | 0.0013 |
| Butyricimonas | 5 | 10.0% | 21.40 | 0.0014 |
| Christensenellaceae R-7 group | 5 | 10.0% | 21.60 | 0.0013 |
| UBA1819 | 5 | 10.0% | 21.80 | 0.0012 |
| Haemophilus | 5 | 10.0% | 25.60 | 0.0008 |
| Monoglobus | 4 | 8.0% | 17.50 | 0.0017 |
| Akkermansia | 4 | 8.0% | 19.75 | 0.0018 |
| Clostridium innocuum group | 4 | 8.0% | 21.00 | 0.0012 |
| NK4A214 group | 4 | 8.0% | 21.50 | 0.0014 |
| Phascolarctobacterium | 4 | 8.0% | 22.75 | 0.0013 |
| Butyricicoccus | 4 | 8.0% | 25.75 | 0.0011 |
| Coprococcus | 3 | 6.0% | 20.33 | 0.0014 |
| Limosilactobacillus | 3 | 6.0% | 22.33 | 0.0010 |
| Ligilactobacillus | 3 | 6.0% | 25.67 | 0.0010 |
| Barnesiella | 3 | 6.0% | 26.00 | 0.0009 |
| Erysipelatoclostridium | 3 | 6.0% | 26.00 | 0.0009 |
| Cloacibacillus | 2 | 4.0% | 16.50 | 0.0024 |
| Dialister | 2 | 4.0% | 21.00 | 0.0015 |
| Others | 2 | 4.0% | 23.00 | 0.0010 |
| Lachnospiraceae NK4A136 group | 2 | 4.0% | 23.50 | 0.0013 |
| Veillonella | 2 | 4.0% | 23.50 | 0.0009 |
| Lachnospiraceae UCG-010 | 2 | 4.0% | 24.50 | 0.0009 |
| Megamonas | 2 | 4.0% | 26.00 | 0.0008 |
| UCG-002 | 2 | 4.0% | 26.50 | 0.0009 |
| Ruminococcus gnavus group | 2 | 4.0% | 27.50 | 0.0010 |
| Anaerostipes | 2 | 4.0% | 28.00 | 0.0009 |
| Escherichia-Shigella | 2 | 4.0% | 29.50 | 0.0008 |
| Lactobacillus | 1 | 2.0% | 22.00 | 0.0010 |
| UCG-005 | 1 | 2.0% | 24.00 | 0.0011 |
| Odoribacter | 1 | 2.0% | 25.00 | 0.0012 |
| Fusobacterium | 1 | 2.0% | 26.00 | 0.0008 |
| Raoultella | 1 | 2.0% | 27.00 | 0.0012 |
| Oscillibacter | 1 | 2.0% | 28.00 | 0.0007 |
| UCG-003 | 1 | 2.0% | 29.00 | 0.0007 |

**Table S6. All adjusted genus-clinical associations** Complete partial Spearman correlation results for 130 genus-clinical variable pairs. Partial Spearman correlations adjust for study group, age, and sex, except that sex is not included as its own covariate when sex is the clinical variable. BH correction was applied across all 130 tests.

| **Genus** | **Clinical variable** | **Partial rho (95% CI)** | **t** | **df** | **P value** | **BH q value** | **Adjustment variables** |
| --- | --- | --- | --- | --- | --- | --- | --- |
| Dorea | Diabetes | 0.334 (0.173, 0.477) | 4.049 | 131 | 8.75e-05 | 0.011 | group;age;sex |
| Bacteroides | Cerebral infarction | -0.292 (-0.441, -0.129) | -3.501 | 131 | 6.34e-04 | 0.041 | group;age;sex |
| Erysipelotrichaceae UCG-003 | Diabetes | 0.241 (0.074, 0.395) | 2.840 | 131 | 0.005 | 0.227 | group;age;sex |
| Flavonifractor | Cerebral infarction | -0.225 (-0.381, -0.057) | -2.642 | 131 | 0.009 | 0.279 | group;age;sex |
| Ruminococcus torques group | Diabetes | 0.221 (0.052, 0.377) | 2.589 | 131 | 0.011 | 0.279 | group;age;sex |
| Eubacterium hallii group | Smoking | 0.203 (0.034, 0.360) | 2.368 | 131 | 0.019 | 0.371 | group;age;sex |
| Christensenellaceae R-7 group | Smoking | 0.196 (0.026, 0.354) | 2.283 | 131 | 0.024 | 0.371 | group;age;sex |
| Eubacterium hallii group | Diabetes | 0.195 (0.025, 0.353) | 2.273 | 131 | 0.025 | 0.371 | group;age;sex |
| Lachnoclostridium | Smoking | -0.193 (-0.352, -0.024) | -2.256 | 131 | 0.026 | 0.371 | group;age;sex |
| Erysipelotrichaceae UCG-003 | Smoking | 0.190 (0.020, 0.349) | 2.215 | 131 | 0.029 | 0.371 | group;age;sex |
| Ruminococcus torques group | Hypertension | 0.185 (0.015, 0.344) | 2.149 | 131 | 0.033 | 0.390 | group;age;sex |
| Bacteroides | Hyperlipidemia | 0.182 (0.012, 0.342) | 2.118 | 131 | 0.036 | 0.390 | group;age;sex |
| Erysipelotrichaceae UCG-003 | Hyperlipidemia | 0.174 (0.004, 0.334) | 2.020 | 131 | 0.045 | 0.413 | group;age;sex |
| Romboutsia | Cerebral infarction | 0.172 (0.002, 0.333) | 2.004 | 131 | 0.047 | 0.413 | group;age;sex |
| Lachnoclostridium | Cerebral infarction | -0.171 (-0.332, -0.001) | -1.986 | 131 | 0.049 | 0.413 | group;age;sex |
| Lachnospira | Smoking | 0.170 (-0.001, 0.330) | 1.971 | 131 | 0.051 | 0.413 | group;age;sex |
| Lachnoclostridium | Alcohol use | -0.165 (-0.326, 0.006) | -1.910 | 131 | 0.058 | 0.441 | group;age;sex |
| Ruminococcus torques group | Coronary heart disease | 0.161 (-0.009, 0.323) | 1.869 | 131 | 0.064 | 0.441 | group;age;sex |
| Blautia | Diabetes | 0.160 (-0.011, 0.321) | 1.853 | 131 | 0.066 | 0.441 | group;age;sex |
| Erysipelotrichaceae UCG-003 | Hypertension | 0.159 (-0.012, 0.320) | 1.841 | 131 | 0.068 | 0.441 | group;age;sex |
| Dorea | Smoking | 0.151 (-0.019, 0.314) | 1.754 | 131 | 0.082 | 0.507 | group;age;sex |
| Erysipelotrichaceae UCG-003 | Coronary heart disease | 0.147 (-0.024, 0.310) | 1.702 | 131 | 0.091 | 0.529 | group;age;sex |
| Lachnoclostridium | Sex | -0.145 (-0.307, 0.025) | -1.689 | 132 | 0.094 | 0.529 | group;age |
| Dorea | Cerebral infarction | 0.144 (-0.027, 0.307) | 1.664 | 131 | 0.099 | 0.534 | group;age;sex |
| Romboutsia | Hypertension | 0.142 (-0.029, 0.305) | 1.644 | 131 | 0.103 | 0.534 | group;age;sex |
| Romboutsia | Alcohol use | -0.138 (-0.301, 0.033) | -1.592 | 131 | 0.114 | 0.569 | group;age;sex |
| Clostridium sensu stricto 1 | Smoking | -0.136 (-0.299, 0.035) | -1.569 | 131 | 0.119 | 0.570 | group;age;sex |
| Clostridium sensu stricto 1 | Cerebral infarction | 0.134 (-0.037, 0.298) | 1.548 | 131 | 0.124 | 0.570 | group;age;sex |
| Christensenellaceae R-7 group | Alcohol use | 0.133 (-0.038, 0.296) | 1.533 | 131 | 0.128 | 0.570 | group;age;sex |
| Romboutsia | Hyperlipidemia | 0.131 (-0.040, 0.295) | 1.517 | 131 | 0.132 | 0.570 | group;age;sex |
| Dorea | Age | -0.126 (-0.289, 0.045) | -1.457 | 132 | 0.147 | 0.618 | group;sex |
| Eubacterium hallii group | COPD | 0.120 (-0.051, 0.285) | 1.385 | 131 | 0.168 | 0.682 | group;age;sex |
| Bacteroides | COPD | -0.119 (-0.283, 0.052) | -1.370 | 131 | 0.173 | 0.682 | group;age;sex |
| Flavonifractor | Age | 0.115 (-0.055, 0.279) | 1.333 | 132 | 0.185 | 0.707 | group;sex |
| Clostridium sensu stricto 1 | Alcohol use | -0.110 (-0.275, 0.061) | -1.266 | 131 | 0.208 | 0.759 | group;age;sex |
| Lachnospira | Age | -0.109 (-0.273, 0.062) | -1.259 | 132 | 0.210 | 0.759 | group;sex |
| Collinsella | Diabetes | 0.106 (-0.065, 0.272) | 1.223 | 131 | 0.223 | 0.785 | group;age;sex |
| Flavonifractor | Smoking | -0.104 (-0.269, 0.068) | -1.193 | 131 | 0.235 | 0.804 | group;age;sex |
| Romboutsia | Diabetes | 0.098 (-0.073, 0.264) | 1.131 | 131 | 0.260 | 0.846 | group;age;sex |
| Dorea | COPD | 0.098 (-0.073, 0.264) | 1.128 | 131 | 0.261 | 0.846 | group;age;sex |
| Christensenellaceae R-7 group | Diabetes | -0.094 (-0.260, 0.077) | -1.081 | 131 | 0.282 | 0.846 | group;age;sex |
| Clostridium sensu stricto 1 | Age | -0.093 (-0.259, 0.078) | -1.075 | 132 | 0.284 | 0.846 | group;sex |
| Blautia | Coronary heart disease | 0.092 (-0.080, 0.258) | 1.055 | 131 | 0.293 | 0.846 | group;age;sex |
| Bacteroides | Age | 0.091 (-0.080, 0.256) | 1.046 | 132 | 0.297 | 0.846 | group;sex |
| Lachnoclostridium | Hypertension | 0.089 (-0.082, 0.256) | 1.027 | 131 | 0.306 | 0.846 | group;age;sex |
| Lachnospira | Alcohol use | 0.089 (-0.082, 0.256) | 1.025 | 131 | 0.307 | 0.846 | group;age;sex |
| Dorea | Coronary heart disease | 0.089 (-0.082, 0.255) | 1.023 | 131 | 0.308 | 0.846 | group;age;sex |
| Flavonifractor | Alcohol use | -0.087 (-0.254, 0.084) | -1.003 | 131 | 0.318 | 0.846 | group;age;sex |
| Collinsella | Sex | -0.086 (-0.252, 0.085) | -0.992 | 132 | 0.323 | 0.846 | group;age |
| Lachnospira | Sex | -0.084 (-0.250, 0.087) | -0.971 | 132 | 0.333 | 0.846 | group;age |
| Lachnospira | Hyperlipidemia | -0.084 (-0.250, 0.088) | -0.961 | 131 | 0.338 | 0.846 | group;age;sex |
| Lachnoclostridium | Age | 0.082 (-0.089, 0.248) | 0.941 | 132 | 0.348 | 0.846 | group;sex |
| Flavonifractor | Hyperlipidemia | -0.081 (-0.248, 0.091) | -0.929 | 131 | 0.355 | 0.846 | group;age;sex |
| Bacteroides | Diabetes | -0.081 (-0.248, 0.091) | -0.927 | 131 | 0.356 | 0.846 | group;age;sex |
| Bacteroides | Alcohol use | -0.078 (-0.245, 0.094) | -0.894 | 131 | 0.373 | 0.846 | group;age;sex |
| Christensenellaceae R-7 group | Hyperlipidemia | -0.076 (-0.243, 0.096) | -0.869 | 131 | 0.387 | 0.846 | group;age;sex |
| Romboutsia | Coronary heart disease | 0.075 (-0.097, 0.242) | 0.859 | 131 | 0.392 | 0.846 | group;age;sex |
| Blautia | Cerebral infarction | 0.074 (-0.098, 0.241) | 0.844 | 131 | 0.400 | 0.846 | group;age;sex |
| Lachnoclostridium | Coronary heart disease | -0.073 (-0.241, 0.098) | -0.841 | 131 | 0.402 | 0.846 | group;age;sex |
| Erysipelotrichaceae UCG-003 | Alcohol use | 0.072 (-0.099, 0.240) | 0.831 | 131 | 0.408 | 0.846 | group;age;sex |
| Ruminococcus torques group | Age | -0.071 (-0.238, 0.099) | -0.822 | 132 | 0.412 | 0.846 | group;sex |
| Erysipelotrichaceae UCG-003 | COPD | 0.071 (-0.100, 0.239) | 0.818 | 131 | 0.415 | 0.846 | group;age;sex |
| Dorea | Alcohol use | 0.069 (-0.103, 0.236) | 0.789 | 131 | 0.431 | 0.846 | group;age;sex |
| Collinsella | Cerebral infarction | 0.069 (-0.103, 0.236) | 0.788 | 131 | 0.432 | 0.846 | group;age;sex |
| Dorea | Sex | -0.068 (-0.235, 0.103) | -0.780 | 132 | 0.437 | 0.846 | group;age |
| Clostridium sensu stricto 1 | Hypertension | 0.067 (-0.105, 0.234) | 0.764 | 131 | 0.446 | 0.846 | group;age;sex |
| Clostridium sensu stricto 1 | COPD | -0.065 (-0.232, 0.107) | -0.743 | 131 | 0.459 | 0.846 | group;age;sex |
| Christensenellaceae R-7 group | Sex | -0.063 (-0.230, 0.107) | -0.730 | 132 | 0.467 | 0.846 | group;age |
| Clostridium sensu stricto 1 | Hyperlipidemia | -0.063 (-0.231, 0.108) | -0.724 | 131 | 0.470 | 0.846 | group;age;sex |
| Eubacterium hallii group | Age | 0.061 (-0.109, 0.229) | 0.707 | 132 | 0.481 | 0.846 | group;sex |
| Ruminococcus torques group | COPD | 0.061 (-0.110, 0.229) | 0.701 | 131 | 0.484 | 0.846 | group;age;sex |
| Flavonifractor | COPD | -0.061 (-0.229, 0.110) | -0.699 | 131 | 0.486 | 0.846 | group;age;sex |
| Clostridium sensu stricto 1 | Sex | 0.060 (-0.110, 0.228) | 0.694 | 132 | 0.489 | 0.846 | group;age |
| Blautia | Age | 0.060 (-0.111, 0.227) | 0.690 | 132 | 0.492 | 0.846 | group;sex |
| Ruminococcus torques group | Hyperlipidemia | 0.059 (-0.112, 0.227) | 0.678 | 131 | 0.499 | 0.846 | group;age;sex |
| Eubacterium hallii group | Hypertension | -0.059 (-0.227, 0.112) | -0.675 | 131 | 0.501 | 0.846 | group;age;sex |
| Blautia | Smoking | 0.059 (-0.113, 0.227) | 0.674 | 131 | 0.501 | 0.846 | group;age;sex |
| Bacteroides | Hypertension | -0.054 (-0.222, 0.118) | -0.615 | 131 | 0.540 | 0.849 | group;age;sex |
| Lachnoclostridium | Diabetes | -0.052 (-0.221, 0.119) | -0.599 | 131 | 0.550 | 0.849 | group;age;sex |
| Clostridium sensu stricto 1 | Coronary heart disease | -0.052 (-0.220, 0.119) | -0.594 | 131 | 0.553 | 0.849 | group;age;sex |
| Christensenellaceae R-7 group | Coronary heart disease | -0.052 (-0.220, 0.120) | -0.591 | 131 | 0.555 | 0.849 | group;age;sex |
| Dorea | Hyperlipidemia | -0.051 (-0.219, 0.120) | -0.584 | 131 | 0.560 | 0.849 | group;age;sex |
| Erysipelotrichaceae UCG-003 | Sex | -0.050 (-0.218, 0.120) | -0.578 | 132 | 0.565 | 0.849 | group;age |
| Eubacterium hallii group | Alcohol use | 0.050 (-0.121, 0.219) | 0.577 | 131 | 0.565 | 0.849 | group;age;sex |
| Lachnospira | Coronary heart disease | -0.050 (-0.218, 0.122) | -0.570 | 131 | 0.570 | 0.849 | group;age;sex |
| Collinsella | Hyperlipidemia | 0.049 (-0.123, 0.217) | 0.556 | 131 | 0.579 | 0.849 | group;age;sex |
| Ruminococcus torques group | Smoking | 0.048 (-0.124, 0.216) | 0.545 | 131 | 0.586 | 0.849 | group;age;sex |
| Eubacterium hallii group | Coronary heart disease | 0.047 (-0.125, 0.215) | 0.534 | 131 | 0.594 | 0.849 | group;age;sex |
| Bacteroides | Sex | -0.046 (-0.214, 0.124) | -0.533 | 132 | 0.595 | 0.849 | group;age |
| Ruminococcus torques group | Cerebral infarction | -0.046 (-0.215, 0.125) | -0.532 | 131 | 0.596 | 0.849 | group;age;sex |
| Collinsella | Age | 0.046 (-0.124, 0.214) | 0.530 | 132 | 0.597 | 0.849 | group;sex |
| Blautia | Sex | -0.046 (-0.214, 0.125) | -0.525 | 132 | 0.601 | 0.849 | group;age |
| Romboutsia | COPD | 0.044 (-0.127, 0.212) | 0.502 | 131 | 0.617 | 0.849 | group;age;sex |
| Bacteroides | Coronary heart disease | -0.044 (-0.212, 0.128) | -0.499 | 131 | 0.619 | 0.849 | group;age;sex |
| Collinsella | Hypertension | -0.043 (-0.212, 0.128) | -0.496 | 131 | 0.620 | 0.849 | group;age;sex |
| Blautia | Hyperlipidemia | -0.042 (-0.211, 0.129) | -0.480 | 131 | 0.632 | 0.852 | group;age;sex |
| Christensenellaceae R-7 group | Age | 0.041 (-0.129, 0.209) | 0.475 | 132 | 0.636 | 0.852 | group;sex |
| Christensenellaceae R-7 group | Cerebral infarction | -0.040 (-0.209, 0.131) | -0.463 | 131 | 0.644 | 0.855 | group;age;sex |
| Romboutsia | Age | -0.039 (-0.208, 0.131) | -0.453 | 132 | 0.651 | 0.855 | group;sex |
| Erysipelotrichaceae UCG-003 | Cerebral infarction | 0.036 (-0.135, 0.205) | 0.417 | 131 | 0.677 | 0.880 | group;age;sex |
| Blautia | COPD | 0.034 (-0.137, 0.203) | 0.387 | 131 | 0.699 | 0.900 | group;age;sex |
| Lachnospira | COPD | 0.033 (-0.138, 0.202) | 0.377 | 131 | 0.707 | 0.901 | group;age;sex |
| Lachnospira | Hypertension | -0.031 (-0.200, 0.140) | -0.358 | 131 | 0.721 | 0.902 | group;age;sex |
| Blautia | Alcohol use | 0.031 (-0.140, 0.200) | 0.358 | 131 | 0.721 | 0.902 | group;age;sex |
| Christensenellaceae R-7 group | COPD | 0.030 (-0.141, 0.199) | 0.345 | 131 | 0.731 | 0.904 | group;age;sex |
| Erysipelotrichaceae UCG-003 | Age | -0.029 (-0.198, 0.141) | -0.334 | 132 | 0.739 | 0.904 | group;sex |
| Romboutsia | Smoking | -0.029 (-0.198, 0.142) | -0.327 | 131 | 0.744 | 0.904 | group;age;sex |
| Christensenellaceae R-7 group | Hypertension | 0.028 (-0.143, 0.197) | 0.315 | 131 | 0.753 | 0.906 | group;age;sex |
| Flavonifractor | Coronary heart disease | 0.026 (-0.145, 0.196) | 0.302 | 131 | 0.763 | 0.910 | group;age;sex |
| Eubacterium hallii group | Sex | -0.022 (-0.191, 0.149) | -0.248 | 132 | 0.804 | 0.944 | group;age |
| Collinsella | Alcohol use | 0.022 (-0.149, 0.191) | 0.246 | 131 | 0.806 | 0.944 | group;age;sex |
| Collinsella | COPD | -0.021 (-0.190, 0.150) | -0.235 | 131 | 0.814 | 0.944 | group;age;sex |
| Lachnospira | Diabetes | 0.019 (-0.151, 0.189) | 0.221 | 131 | 0.825 | 0.944 | group;age;sex |
| Flavonifractor | Hypertension | -0.019 (-0.189, 0.152) | -0.218 | 131 | 0.828 | 0.944 | group;age;sex |
| Bacteroides | Smoking | -0.017 (-0.187, 0.154) | -0.195 | 131 | 0.846 | 0.951 | group;age;sex |
| Ruminococcus torques group | Sex | -0.016 (-0.185, 0.154) | -0.184 | 132 | 0.854 | 0.951 | group;age |
| Dorea | Hypertension | -0.015 (-0.184, 0.156) | -0.168 | 131 | 0.867 | 0.951 | group;age;sex |
| Blautia | Hypertension | 0.014 (-0.156, 0.184) | 0.161 | 131 | 0.872 | 0.951 | group;age;sex |
| Flavonifractor | Sex | 0.013 (-0.157, 0.182) | 0.151 | 132 | 0.880 | 0.951 | group;age |
| Clostridium sensu stricto 1 | Diabetes | -0.013 (-0.183, 0.157) | -0.150 | 131 | 0.881 | 0.951 | group;age;sex |
| Eubacterium hallii group | Hyperlipidemia | -0.012 (-0.182, 0.158) | -0.142 | 131 | 0.887 | 0.951 | group;age;sex |
| Collinsella | Coronary heart disease | -0.010 (-0.180, 0.160) | -0.120 | 131 | 0.905 | 0.951 | group;age;sex |
| Romboutsia | Sex | -0.010 (-0.179, 0.160) | -0.117 | 132 | 0.907 | 0.951 | group;age |
| Lachnoclostridium | Hyperlipidemia | -0.009 (-0.179, 0.161) | -0.107 | 131 | 0.915 | 0.951 | group;age;sex |
| Lachnospira | Cerebral infarction | -0.009 (-0.179, 0.162) | -0.101 | 131 | 0.920 | 0.951 | group;age;sex |
| Collinsella | Smoking | 0.009 (-0.162, 0.179) | 0.098 | 131 | 0.922 | 0.951 | group;age;sex |
| Flavonifractor | Diabetes | -0.005 (-0.175, 0.166) | -0.053 | 131 | 0.957 | 0.980 | group;age;sex |
| Lachnoclostridium | COPD | 0.003 (-0.167, 0.174) | 0.039 | 131 | 0.969 | 0.984 | group;age;sex |
| Eubacterium hallii group | Cerebral infarction | 0.002 (-0.168, 0.173) | 0.028 | 131 | 0.978 | 0.985 | group;age;sex |
| Ruminococcus torques group | Alcohol use | -0.002 (-0.172, 0.169) | -0.018 | 131 | 0.986 | 0.986 | group;age;sex |

Table S7. Additional clinical descriptors in participants with CSDH

| **Characteristic** | **CSDH** |
| --- | --- |
| Participants | 40 |
| Total hematoma volume, mL | 111.77 (82.36-127.53) |
| Midline shift, mm | 8.77 (4.82-11.23) |
| Homogeneous architecture | 18 (45.0%) |
| Laminar architecture | 2 (5.0%) |
| Separated architecture | 4 (10.0%) |
| Trabecular architecture | 15 (37.5%) |
| Mixed bilateral architecture | 1 (2.5%) |
| Postoperative recurrence within 180 days | 1 (2.5%) |

Continuous measures are median (interquartile range); categorical variables are n (%). Volume was measured by slice-by-slice segmentation in 3D Slicer, with bilateral volumes summed. Architecture is reported per participant; the mixed bilateral category comprises one participant with a trabecular right-sided and homogeneous left-sided hematoma. Architecture describes the recorded hematoma classification and is not a separate quantitative assessment of membrane septations. These variables are not applicable to controls. Volume and midline-shift summaries include all 40 cases. Source volumes in mm³ were divided by 1,000 to obtain mL. Recurrence refers to events recorded within 180 days after surgery, not disease status at enrollment; it is reported descriptively and was not included in baseline covariate adjustment.

Table S8. Genus-level sensitivity analyses with additional diabetes and medication adjustment

| **Genus** | **Diabetes-adjusted coefficient (95% CI)** | **BH q** | **Diabetes and medication-adjusted coefficient (95% CI)** | **BH q** |
| --- | --- | --- | --- | --- |
| Acinetobacter | 0.083 (-0.590 to 0.755) | 0.904 | 0.033 (-0.646 to 0.713) | 0.952 |
| Agathobacter | 0.292 (-0.831 to 1.415) | 0.815 | 0.255 (-0.883 to 1.393) | 0.835 |
| Akkermansia | -0.827 (-2.202 to 0.549) | 0.467 | -0.562 (-1.914 to 0.791) | 0.637 |
| Alistipes | -0.136 (-1.284 to 1.012) | 0.904 | -0.148 (-1.318 to 1.022) | 0.931 |
| Anaerostipes | 0.446 (-0.290 to 1.182) | 0.467 | 0.417 (-0.326 to 1.161) | 0.527 |
| Bacteroides | -1.940 (-2.466 to -1.414) | <0.001 | -1.915 (-2.449 to -1.382) | <0.001 |
| Barnesiella | 0.253 (-0.972 to 1.479) | 0.878 | 0.315 (-0.932 to 1.561) | 0.821 |
| Bifidobacterium | 0.416 (-0.634 to 1.466) | 0.642 | 0.399 (-0.671 to 1.470) | 0.656 |
| Blautia | 1.083 (0.600 to 1.567) | <0.001 | 1.115 (0.627 to 1.603) | <0.001 |
| Butyricicoccus | -0.012 (-0.639 to 0.615) | 0.984 | 0.030 (-0.607 to 0.668) | 0.952 |
| Butyricimonas | 0.049 (-0.988 to 1.086) | 0.981 | 0.129 (-0.904 to 1.163) | 0.931 |
| CAG-56 | 0.515 (-0.533 to 1.563) | 0.549 | 0.564 (-0.499 to 1.627) | 0.538 |
| Christensenellaceae R-7 group | 1.837 (0.701 to 2.972) | 0.012 | 1.793 (0.662 to 2.923) | 0.015 |
| Citrobacter | 0.265 (-0.682 to 1.213) | 0.793 | 0.225 (-0.732 to 1.181) | 0.830 |
| Clostridium innocuum group | -0.362 (-1.188 to 0.465) | 0.586 | -0.395 (-1.231 to 0.441) | 0.584 |
| Clostridium sensu stricto 1 | 1.532 (0.537 to 2.528) | 0.018 | 1.466 (0.459 to 2.473) | 0.028 |
| Collinsella | 2.998 (1.854 to 4.141) | <0.001 | 2.937 (1.787 to 4.088) | <0.001 |
| Coprococcus | 0.946 (0.081 to 1.810) | 0.153 | 0.976 (0.096 to 1.856) | 0.142 |
| Dialister | 1.098 (-0.281 to 2.476) | 0.309 | 1.083 (-0.317 to 2.482) | 0.337 |
| Dorea | 2.054 (1.302 to 2.807) | <0.001 | 2.029 (1.268 to 2.790) | <0.001 |
| Enterobacter | 0.223 (-0.416 to 0.862) | 0.697 | 0.195 (-0.456 to 0.846) | 0.756 |
| Enterococcus | -0.990 (-2.049 to 0.069) | 0.220 | -0.952 (-2.031 to 0.127) | 0.273 |
| Erysipelatoclostridium | -0.130 (-0.904 to 0.645) | 0.904 | -0.191 (-0.963 to 0.580) | 0.821 |
| Erysipelotrichaceae UCG-003 | 1.914 (1.068 to 2.760) | <0.001 | 1.924 (1.062 to 2.786) | <0.001 |
| Escherichia-Shigella | 0.631 (-0.466 to 1.729) | 0.493 | 0.551 (-0.558 to 1.659) | 0.567 |
| Eubacterium eligens group | -0.593 (-1.684 to 0.499) | 0.518 | -0.498 (-1.602 to 0.607) | 0.591 |
| Eubacterium hallii group | 1.412 (0.610 to 2.214) | 0.007 | 1.333 (0.528 to 2.138) | 0.011 |
| Eubacterium ruminantium group | -0.252 (-1.318 to 0.814) | 0.842 | -0.085 (-1.117 to 0.947) | 0.951 |
| Eubacterium ventriosum group | 0.118 (-0.805 to 1.041) | 0.904 | 0.110 (-0.832 to 1.051) | 0.931 |
| Faecalibacterium | 0.062 (-0.968 to 1.092) | 0.974 | 0.112 (-0.933 to 1.158) | 0.931 |
| Flavonifractor | -1.456 (-2.229 to -0.684) | 0.003 | -1.416 (-2.198 to -0.634) | 0.006 |
| Fusicatenibacter | 0.650 (-0.283 to 1.584) | 0.418 | 0.674 (-0.277 to 1.625) | 0.413 |
| Fusobacterium | 0.435 (-0.516 to 1.385) | 0.576 | 0.445 (-0.517 to 1.407) | 0.584 |
| Haemophilus | -0.116 (-1.029 to 0.798) | 0.904 | -0.095 (-1.020 to 0.830) | 0.931 |
| Holdemanella | 0.897 (-0.067 to 1.860) | 0.220 | 0.841 (-0.134 to 1.815) | 0.273 |
| Intestinibacter | 0.885 (-0.038 to 1.807) | 0.220 | 0.796 (-0.133 to 1.725) | 0.273 |
| Klebsiella | 0.252 (-1.004 to 1.508) | 0.878 | 0.272 (-1.007 to 1.550) | 0.841 |
| Lachnoclostridium | -0.961 (-1.605 to -0.317) | 0.021 | -0.946 (-1.602 to -0.290) | 0.028 |
| Lachnospira | -1.525 (-2.531 to -0.519) | 0.019 | -1.572 (-2.596 to -0.548) | 0.019 |
| Lachnospiraceae ND3007 group | -0.694 (-1.543 to 0.154) | 0.299 | -0.742 (-1.604 to 0.121) | 0.273 |
| Lachnospiraceae NK4A136 group | 0.147 (-0.821 to 1.115) | 0.904 | 0.319 (-0.640 to 1.279) | 0.712 |
| Lactobacillus | 0.563 (-0.271 to 1.398) | 0.421 | 0.555 (-0.293 to 1.403) | 0.453 |
| Lactonifactor | 0.513 (-0.234 to 1.259) | 0.418 | 0.513 (-0.246 to 1.271) | 0.434 |
| Ligilactobacillus | 0.308 (-0.789 to 1.405) | 0.793 | 0.422 (-0.688 to 1.531) | 0.656 |
| Limosilactobacillus | 0.139 (-0.885 to 1.164) | 0.904 | 0.068 (-0.972 to 1.109) | 0.952 |
| Megamonas | -0.529 (-1.701 to 0.643) | 0.576 | -0.469 (-1.651 to 0.713) | 0.655 |
| Megasphaera | -0.548 (-1.575 to 0.480) | 0.521 | -0.662 (-1.699 to 0.375) | 0.464 |
| Monoglobus | 0.708 (-0.058 to 1.474) | 0.220 | 0.722 (-0.055 to 1.499) | 0.270 |
| NK4A214 group | 0.685 (-0.455 to 1.824) | 0.467 | 0.622 (-0.523 to 1.767) | 0.531 |
| Olsenella | 0.945 (0.204 to 1.686) | 0.065 | 0.862 (0.115 to 1.610) | 0.123 |
| Oscillibacter | -0.006 (-0.843 to 0.830) | 0.989 | 0.048 (-0.803 to 0.899) | 0.952 |
| Others | 0.030 (-0.391 to 0.451) | 0.971 | 0.050 (-0.375 to 0.476) | 0.931 |
| Parabacteroides | -0.413 (-1.228 to 0.403) | 0.539 | -0.385 (-1.216 to 0.445) | 0.584 |
| Parasutterella | -1.040 (-2.171 to 0.092) | 0.220 | -1.015 (-2.167 to 0.137) | 0.273 |
| Phascolarctobacterium | -0.494 (-1.781 to 0.793) | 0.650 | -0.485 (-1.775 to 0.804) | 0.656 |
| Prevotella 9 | 0.692 (-0.771 to 2.155) | 0.567 | 0.741 (-0.746 to 2.228) | 0.567 |
| Raoultella | 0.149 (-0.800 to 1.099) | 0.904 | 0.120 (-0.848 to 1.088) | 0.931 |
| Romboutsia | 1.730 (0.731 to 2.728) | 0.007 | 1.731 (0.725 to 2.736) | 0.008 |
| Roseburia | 0.579 (-0.555 to 1.713) | 0.539 | 0.650 (-0.502 to 1.803) | 0.527 |
| Ruminococcus | 1.047 (-0.045 to 2.138) | 0.220 | 1.159 (0.054 to 2.264) | 0.177 |
| Ruminococcus gnavus group | -0.034 (-1.183 to 1.116) | 0.981 | -0.028 (-1.181 to 1.126) | 0.976 |
| Ruminococcus torques group | 1.303 (0.529 to 2.077) | 0.009 | 1.347 (0.561 to 2.132) | 0.008 |
| Sellimonas | -0.575 (-1.517 to 0.366) | 0.467 | -0.532 (-1.486 to 0.423) | 0.527 |
| Streptococcus | 0.877 (0.023 to 1.732) | 0.197 | 0.887 (0.025 to 1.748) | 0.183 |
| Subdoligranulum | 0.036 (-1.050 to 1.122) | 0.981 | 0.006 (-1.085 to 1.097) | 0.991 |
| Sutterella | -0.814 (-1.758 to 0.129) | 0.266 | -0.792 (-1.749 to 0.164) | 0.295 |
| UBA1819 | -0.495 (-1.271 to 0.280) | 0.462 | -0.472 (-1.262 to 0.319) | 0.516 |
| UCG-002 | 1.167 (-0.101 to 2.435) | 0.220 | 1.151 (-0.132 to 2.434) | 0.273 |
| UCG-003 | 0.489 (-0.374 to 1.352) | 0.494 | 0.485 (-0.390 to 1.361) | 0.527 |
| UCG-005 | 0.884 (-0.201 to 1.968) | 0.299 | 0.876 (-0.213 to 1.965) | 0.311 |
| Veillonella | -0.756 (-1.824 to 0.313) | 0.416 | -0.724 (-1.771 to 0.323) | 0.425 |

All 71 eligible genera are shown; n = 136 in both models. Both models include disease group, age, sex, hypertension, smoking and alcohol use. The first additionally includes diabetes; the second also includes statin, anticoagulant and antiplatelet exposure. Positive coefficients indicate higher CSDH CLR abundance relative to the within-sample geometric mean across 344 genera. BH adjustment was applied to 71 tests separately in each model. The original rarefied counts have a common depth of 46,530. Full estimates and p values are available in CLR_all_genera_all_models.csv.

Table S9. Genus-level sensitivity analyses using non-rarefied counts

| **Genus** | **Original model coefficient (95% CI)** | **BH q** | **Add diabetes coefficient (95% CI)** | **BH q** | **Add diabetes and medications coefficient (95% CI)** | **BH q** |
| --- | --- | --- | --- | --- | --- | --- |
| Acinetobacter | 0.154 (-0.646 to 0.954) | 0.860 | 0.251 (-0.562 to 1.064) | 0.727 | 0.201 (-0.624 to 1.026) | 0.800 |
| Agathobacter | 0.367 (-0.795 to 1.528) | 0.728 | 0.323 (-0.864 to 1.510) | 0.777 | 0.273 (-0.930 to 1.475) | 0.804 |
| Akkermansia | -0.707 (-2.236 to 0.822) | 0.584 | -1.003 (-2.542 to 0.536) | 0.452 | -0.710 (-2.223 to 0.803) | 0.574 |
| Alistipes | -0.241 (-1.486 to 1.005) | 0.860 | -0.177 (-1.449 to 1.095) | 0.880 | -0.192 (-1.489 to 1.104) | 0.882 |
| Anaerostipes | 0.554 (-0.210 to 1.318) | 0.364 | 0.498 (-0.281 to 1.278) | 0.452 | 0.473 (-0.314 to 1.259) | 0.489 |
| Bacteroides | -2.026 (-2.546 to -1.507) | <0.001 | -1.978 (-2.507 to -1.449) | <0.001 | -1.953 (-2.489 to -1.416) | <0.001 |
| Barnesiella | 0.254 (-1.135 to 1.643) | 0.860 | 0.305 (-1.114 to 1.724) | 0.823 | 0.377 (-1.066 to 1.820) | 0.792 |
| Bifidobacterium | 0.369 (-0.694 to 1.431) | 0.694 | 0.372 (-0.715 to 1.458) | 0.682 | 0.362 (-0.746 to 1.470) | 0.703 |
| Blautia | 1.130 (0.648 to 1.611) | <0.001 | 1.045 (0.559 to 1.531) | <0.001 | 1.077 (0.587 to 1.568) | <0.001 |
| Butyricicoccus | 0.095 (-0.599 to 0.789) | 0.860 | -0.019 (-0.721 to 0.683) | 0.976 | 0.036 (-0.677 to 0.748) | 0.956 |
| Butyricimonas | 0.052 (-1.141 to 1.244) | 0.967 | 0.206 (-1.005 to 1.417) | 0.869 | 0.309 (-0.900 to 1.518) | 0.792 |
| CAG-56 | 0.853 (-0.342 to 2.047) | 0.367 | 0.768 (-0.451 to 1.987) | 0.452 | 0.825 (-0.412 to 2.062) | 0.470 |
| Christensenellaceae R-7 group | 1.804 (0.538 to 3.069) | 0.028 | 1.929 (0.640 to 3.218) | 0.023 | 1.869 (0.591 to 3.147) | 0.028 |
| Citrobacter | 0.405 (-0.665 to 1.476) | 0.673 | 0.446 (-0.649 to 1.540) | 0.611 | 0.413 (-0.695 to 1.522) | 0.656 |
| Clostridium innocuum group | -0.393 (-1.331 to 0.546) | 0.618 | -0.458 (-1.416 to 0.500) | 0.535 | -0.487 (-1.455 to 0.482) | 0.572 |
| Clostridium sensu stricto 1 | 1.701 (0.604 to 2.799) | 0.019 | 1.734 (0.612 to 2.856) | 0.019 | 1.657 (0.523 to 2.792) | 0.028 |
| Collinsella | 3.342 (2.056 to 4.627) | <0.001 | 3.192 (1.885 to 4.499) | <0.001 | 3.128 (1.812 to 4.443) | <0.001 |
| Coprococcus | 1.058 (0.143 to 1.974) | 0.113 | 0.980 (0.047 to 1.914) | 0.176 | 1.012 (0.062 to 1.963) | 0.175 |
| Dialister | 1.105 (-0.347 to 2.558) | 0.356 | 1.215 (-0.266 to 2.697) | 0.316 | 1.204 (-0.300 to 2.708) | 0.333 |
| Dorea | 2.377 (1.531 to 3.222) | <0.001 | 2.178 (1.333 to 3.022) | <0.001 | 2.150 (1.297 to 3.004) | <0.001 |
| Enterobacter | 0.281 (-0.499 to 1.062) | 0.691 | 0.399 (-0.392 to 1.189) | 0.533 | 0.375 (-0.429 to 1.179) | 0.574 |
| Enterococcus | -0.975 (-2.174 to 0.224) | 0.343 | -1.104 (-2.324 to 0.116) | 0.256 | -1.062 (-2.305 to 0.180) | 0.328 |
| Erysipelatoclostridium | 0.128 (-0.789 to 1.046) | 0.860 | -0.038 (-0.964 to 0.888) | 0.976 | -0.108 (-1.034 to 0.818) | 0.893 |
| Erysipelotrichaceae UCG-003 | 2.364 (1.363 to 3.365) | <0.001 | 2.156 (1.151 to 3.161) | <0.001 | 2.165 (1.140 to 3.189) | <0.001 |
| Escherichia-Shigella | 0.713 (-0.396 to 1.823) | 0.411 | 0.615 (-0.515 to 1.746) | 0.515 | 0.539 (-0.603 to 1.682) | 0.574 |
| Eubacterium eligens group | -0.562 (-1.772 to 0.648) | 0.584 | -0.597 (-1.834 to 0.640) | 0.535 | -0.500 (-1.754 to 0.754) | 0.626 |
| Eubacterium hallii group | 1.682 (0.791 to 2.573) | 0.003 | 1.472 (0.582 to 2.362) | 0.012 | 1.390 (0.493 to 2.286) | 0.021 |
| Eubacterium ruminantium group | -0.172 (-1.385 to 1.042) | 0.860 | -0.270 (-1.508 to 0.967) | 0.823 | -0.070 (-1.269 to 1.130) | 0.956 |
| Eubacterium ventriosum group | 0.227 (-0.789 to 1.244) | 0.851 | 0.170 (-0.868 to 1.208) | 0.869 | 0.168 (-0.890 to 1.226) | 0.881 |
| Faecalibacterium | -0.135 (-1.204 to 0.934) | 0.864 | -0.011 (-1.098 to 1.076) | 0.984 | 0.038 (-1.065 to 1.141) | 0.956 |
| Flavonifractor | -1.640 (-2.474 to -0.805) | 0.002 | -1.637 (-2.490 to -0.783) | 0.003 | -1.588 (-2.453 to -0.724) | 0.005 |
| Fusicatenibacter | 0.804 (-0.178 to 1.787) | 0.343 | 0.783 (-0.222 to 1.787) | 0.330 | 0.803 (-0.220 to 1.825) | 0.333 |
| Fusobacterium | 0.544 (-0.551 to 1.639) | 0.567 | 0.481 (-0.637 to 1.600) | 0.586 | 0.489 (-0.642 to 1.620) | 0.595 |
| Haemophilus | -0.289 (-1.352 to 0.773) | 0.792 | -0.180 (-1.262 to 0.902) | 0.869 | -0.171 (-1.263 to 0.921) | 0.881 |
| Holdemanella | 0.898 (-0.209 to 2.004) | 0.343 | 1.053 (-0.070 to 2.176) | 0.248 | 0.983 (-0.150 to 2.117) | 0.328 |
| Intestinibacter | 0.943 (-0.070 to 1.955) | 0.253 | 0.968 (-0.067 to 2.003) | 0.248 | 0.868 (-0.173 to 1.909) | 0.328 |
| Klebsiella | 0.212 (-1.153 to 1.577) | 0.860 | 0.311 (-1.082 to 1.704) | 0.823 | 0.319 (-1.097 to 1.734) | 0.804 |
| Lachnoclostridium | -1.003 (-1.671 to -0.335) | 0.021 | -0.999 (-1.682 to -0.316) | 0.025 | -0.979 (-1.674 to -0.284) | 0.033 |
| Lachnospira | -1.618 (-2.730 to -0.505) | 0.026 | -1.618 (-2.756 to -0.481) | 0.029 | -1.679 (-2.835 to -0.523) | 0.028 |
| Lachnospiraceae ND3007 group | -0.562 (-1.530 to 0.406) | 0.460 | -0.550 (-1.540 to 0.439) | 0.515 | -0.601 (-1.607 to 0.406) | 0.489 |
| Lachnospiraceae NK4A136 group | 0.175 (-0.899 to 1.249) | 0.860 | 0.160 (-0.938 to 1.258) | 0.880 | 0.351 (-0.738 to 1.440) | 0.703 |
| Lactobacillus | 0.632 (-0.356 to 1.620) | 0.411 | 0.786 (-0.215 to 1.786) | 0.330 | 0.789 (-0.226 to 1.804) | 0.333 |
| Lactonifactor | 0.687 (-0.213 to 1.586) | 0.356 | 0.439 (-0.452 to 1.329) | 0.535 | 0.441 (-0.464 to 1.346) | 0.574 |
| Ligilactobacillus | 0.281 (-0.963 to 1.524) | 0.851 | 0.456 (-0.804 to 1.717) | 0.661 | 0.587 (-0.688 to 1.862) | 0.574 |
| Limosilactobacillus | -0.047 (-1.227 to 1.133) | 0.967 | 0.149 (-1.044 to 1.342) | 0.880 | 0.052 (-1.157 to 1.262) | 0.956 |
| Megamonas | -0.448 (-1.753 to 0.857) | 0.694 | -0.506 (-1.839 to 0.827) | 0.645 | -0.435 (-1.779 to 0.910) | 0.703 |
| Megasphaera | -0.871 (-2.066 to 0.324) | 0.364 | -0.629 (-1.830 to 0.572) | 0.523 | -0.780 (-1.989 to 0.429) | 0.482 |
| Monoglobus | 0.969 (0.120 to 1.819) | 0.114 | 0.914 (0.047 to 1.781) | 0.176 | 0.918 (0.039 to 1.798) | 0.175 |
| NK4A214 group | 0.831 (-0.455 to 2.116) | 0.411 | 0.825 (-0.489 to 2.140) | 0.452 | 0.751 (-0.570 to 2.072) | 0.501 |
| Olsenella | 1.277 (0.429 to 2.124) | 0.021 | 1.286 (0.420 to 2.153) | 0.023 | 1.194 (0.318 to 2.069) | 0.040 |
| Oscillibacter | -0.017 (-0.948 to 0.915) | 0.972 | 0.062 (-0.888 to 1.011) | 0.952 | 0.129 (-0.836 to 1.095) | 0.892 |
| Others | 0.015 (-0.390 to 0.420) | 0.967 | -0.010 (-0.423 to 0.403) | 0.976 | 0.012 (-0.406 to 0.430) | 0.956 |
| Parabacteroides | -0.455 (-1.309 to 0.399) | 0.521 | -0.438 (-1.311 to 0.435) | 0.533 | -0.400 (-1.288 to 0.487) | 0.577 |
| Parasutterella | -0.959 (-2.227 to 0.308) | 0.356 | -1.080 (-2.371 to 0.211) | 0.310 | -1.029 (-2.344 to 0.286) | 0.333 |
| Phascolarctobacterium | -0.588 (-1.995 to 0.818) | 0.618 | -0.618 (-2.056 to 0.819) | 0.586 | -0.602 (-2.039 to 0.835) | 0.604 |
| Prevotella 9 | 0.681 (-0.929 to 2.292) | 0.618 | 0.878 (-0.758 to 2.514) | 0.515 | 0.932 (-0.730 to 2.595) | 0.501 |
| Raoultella | 0.227 (-0.852 to 1.305) | 0.860 | 0.236 (-0.866 to 1.339) | 0.823 | 0.213 (-0.913 to 1.338) | 0.853 |
| Romboutsia | 1.920 (0.826 to 3.015) | 0.006 | 1.824 (0.708 to 2.940) | 0.012 | 1.811 (0.689 to 2.933) | 0.016 |
| Roseburia | 0.730 (-0.472 to 1.933) | 0.445 | 0.675 (-0.554 to 1.903) | 0.515 | 0.744 (-0.506 to 1.995) | 0.489 |
| Ruminococcus | 1.120 (-0.043 to 2.282) | 0.232 | 1.137 (-0.052 to 2.325) | 0.248 | 1.251 (0.046 to 2.455) | 0.175 |
| Ruminococcus gnavus group | 0.032 (-1.240 to 1.304) | 0.972 | -0.164 (-1.452 to 1.124) | 0.880 | -0.161 (-1.451 to 1.129) | 0.893 |
| Ruminococcus torques group | 1.493 (0.686 to 2.299) | 0.003 | 1.327 (0.516 to 2.137) | 0.012 | 1.365 (0.542 to 2.188) | 0.014 |
| Sellimonas | -0.649 (-1.754 to 0.456) | 0.460 | -0.683 (-1.813 to 0.446) | 0.473 | -0.634 (-1.780 to 0.511) | 0.501 |
| Streptococcus | 0.847 (-0.012 to 1.707) | 0.223 | 0.811 (-0.067 to 1.690) | 0.249 | 0.821 (-0.065 to 1.708) | 0.273 |
| Subdoligranulum | -0.161 (-1.333 to 1.010) | 0.860 | -0.080 (-1.276 to 1.116) | 0.952 | -0.116 (-1.317 to 1.086) | 0.914 |
| Sutterella | -0.907 (-2.014 to 0.200) | 0.343 | -0.787 (-1.914 to 0.339) | 0.429 | -0.758 (-1.901 to 0.385) | 0.470 |
| UBA1819 | -0.550 (-1.378 to 0.277) | 0.410 | -0.560 (-1.406 to 0.286) | 0.452 | -0.528 (-1.391 to 0.335) | 0.489 |
| UCG-002 | 1.076 (-0.292 to 2.445) | 0.356 | 1.204 (-0.190 to 2.599) | 0.290 | 1.179 (-0.231 to 2.588) | 0.328 |
| UCG-003 | 0.469 (-0.533 to 1.471) | 0.584 | 0.583 (-0.437 to 1.602) | 0.513 | 0.586 (-0.447 to 1.618) | 0.501 |
| UCG-005 | 0.901 (-0.301 to 2.103) | 0.356 | 0.994 (-0.232 to 2.220) | 0.316 | 0.985 (-0.243 to 2.214) | 0.333 |
| Veillonella | -0.817 (-1.977 to 0.344) | 0.369 | -0.763 (-1.949 to 0.423) | 0.452 | -0.734 (-1.894 to 0.425) | 0.487 |

All 71 eligible genera are shown; n = 136 in each model. The original model adjusts for age, sex, hypertension, smoking and alcohol use. The diabetes model additionally adjusts for diabetes; the medication-expanded model additionally includes diabetes and statin, anticoagulant and antiplatelet exposure. Genus counts were divided by participant-level total counts (range, 46,530-139,003), then processed using the same half-minimum pseudocount rule, 344-genus CLR and filtering criteria. BH adjustment was applied to all 71 tests separately in each model. All 13 primary associations persisted, with Olsenella additionally significant in these non-rarefied analyses. Full estimates and p values are available in CLR_all_genera_all_models.csv.

Table S10. Exploratory bootstrap stability of the 13 primary genus associations

| **Model** | **Genus** | **Coefficient** | **Bootstrap 95% interval** | **Valid replicates** | **Same direction** |
| --- | --- | --- | --- | --- | --- |
| Original | Bacteroides | -1.988 | -2.569 to -1.394 | 1000 | 100.0% |
| Original | Collinsella | 3.134 | 2.037 to 4.278 | 1000 | 100.0% |
| Original | Blautia | 1.169 | 0.746 to 1.615 | 1000 | 100.0% |
| Original | Ruminococcus torques group | 1.463 | 0.755 to 2.182 | 1000 | 100.0% |
| Original | Clostridium sensu stricto 1 | 1.507 | 0.471 to 2.453 | 1000 | 99.7% |
| Original | Christensenellaceae R-7 group | 1.722 | 0.600 to 2.820 | 1000 | 100.0% |
| Original | Lachnoclostridium | -0.969 | -1.578 to -0.378 | 1000 | 100.0% |
| Original | Erysipelotrichaceae UCG-003 | 2.106 | 1.342 to 2.930 | 1000 | 100.0% |
| Original | Romboutsia | 1.817 | 0.818 to 2.821 | 1000 | 100.0% |
| Original | Lachnospira | -1.521 | -2.416 to -0.578 | 1000 | 99.9% |
| Original | Dorea | 2.251 | 1.633 to 2.987 | 1000 | 100.0% |
| Original | Eubacterium hallii group | 1.600 | 0.930 to 2.420 | 1000 | 100.0% |
| Original | Flavonifractor | -1.442 | -2.180 to -0.716 | 1000 | 99.9% |
| Add diabetes | Bacteroides | -1.940 | -2.508 to -1.327 | 1000 | 100.0% |
| Add diabetes | Collinsella | 2.998 | 1.906 to 4.171 | 1000 | 100.0% |
| Add diabetes | Blautia | 1.083 | 0.657 to 1.529 | 1000 | 100.0% |
| Add diabetes | Ruminococcus torques group | 1.303 | 0.626 to 1.977 | 1000 | 100.0% |
| Add diabetes | Clostridium sensu stricto 1 | 1.532 | 0.471 to 2.494 | 1000 | 99.6% |
| Add diabetes | Christensenellaceae R-7 group | 1.837 | 0.674 to 2.987 | 1000 | 100.0% |
| Add diabetes | Lachnoclostridium | -0.961 | -1.587 to -0.343 | 1000 | 100.0% |
| Add diabetes | Erysipelotrichaceae UCG-003 | 1.914 | 1.153 to 2.715 | 1000 | 100.0% |
| Add diabetes | Romboutsia | 1.730 | 0.692 to 2.721 | 1000 | 100.0% |
| Add diabetes | Lachnospira | -1.525 | -2.479 to -0.595 | 1000 | 99.8% |
| Add diabetes | Dorea | 2.054 | 1.444 to 2.792 | 1000 | 100.0% |
| Add diabetes | Eubacterium hallii group | 1.412 | 0.759 to 2.218 | 1000 | 100.0% |
| Add diabetes | Flavonifractor | -1.456 | -2.242 to -0.753 | 1000 | 100.0% |
| Add diabetes and medications | Bacteroides | -1.915 | -2.524 to -1.313 | 996 | 100.0% |
| Add diabetes and medications | Collinsella | 2.937 | 1.788 to 4.091 | 996 | 100.0% |
| Add diabetes and medications | Blautia | 1.115 | 0.684 to 1.591 | 996 | 100.0% |
| Add diabetes and medications | Ruminococcus torques group | 1.347 | 0.642 to 2.063 | 996 | 100.0% |
| Add diabetes and medications | Clostridium sensu stricto 1 | 1.466 | 0.404 to 2.475 | 996 | 99.6% |
| Add diabetes and medications | Christensenellaceae R-7 group | 1.793 | 0.599 to 2.960 | 996 | 100.0% |
| Add diabetes and medications | Lachnoclostridium | -0.946 | -1.600 to -0.346 | 996 | 99.8% |
| Add diabetes and medications | Erysipelotrichaceae UCG-003 | 1.924 | 1.138 to 2.773 | 996 | 100.0% |
| Add diabetes and medications | Romboutsia | 1.731 | 0.653 to 2.729 | 996 | 100.0% |
| Add diabetes and medications | Lachnospira | -1.572 | -2.498 to -0.592 | 996 | 99.9% |
| Add diabetes and medications | Dorea | 2.029 | 1.373 to 2.811 | 996 | 100.0% |
| Add diabetes and medications | Eubacterium hallii group | 1.333 | 0.691 to 2.146 | 996 | 100.0% |
| Add diabetes and medications | Flavonifractor | -1.416 | -2.229 to -0.672 | 996 | 99.9% |

Bootstrap resampling used 1,000 attempts within disease groups, retaining 40 cases and 96 controls. The original 13 genera and the CLR transformation were held fixed. Four medication-expanded replicates were rank deficient and omitted. Intervals are pointwise percentile intervals conditional on the original selection; they are not adjusted for multiple testing or feature selection. Direction retention is calculated among valid replicates. These summaries assess internal stability and do not demonstrate adequate power, absence of residual confounding or external validation.
